# Non-Redundant tRNA Reference Sequences for Deep Sequencing Analysis of tRNA Abundance and Epitranscriptomic RNA Modifications

**DOI:** 10.3390/genes12010081

**Published:** 2021-01-10

**Authors:** Florian PICHOT, Virginie MARCHAND, Mark HELM, Yuri MOTORIN

**Affiliations:** 1 Institute of Pharmaceutical and Biomedical Sciences, Johannes Gutenberg-University Mainz, Staudingerweg 5, 55128 Mainz, Germany; fpichot@uni-mainz.de (F.P.); mhelm@uni-mainz.de (M.H.); 2 Université de Lorraine, CNRS, INSERM, IBSLor (UMS2008/US40), Epitranscriptomics and RNA Sequencing Core Facility, F54000 Nancy, France; Virginie.Marchand@univ-lorraine.fr; 3 Université de Lorraine, CNRS, IMoPA (UMR7365), F54000 Nancy, France

**Keywords:** tRNA, reference sequence, deep sequencing, RNA modifications, epitranscriptome, tRNA pool, quantification

## Abstract

Analysis of RNA by deep-sequencing approaches has found widespread application in modern biology. In addition to measurements of RNA abundance under various physiological conditions, such techniques are now widely used for mapping and quantification of RNA modifications. Transfer RNA (tRNA) molecules are among the frequent targets of such investigation, since they contain multiple modified residues. However, the major challenge in tRNA examination is related to a large number of duplicated and point-mutated genes encoding those RNA molecules. Moreover, the existence of multiple isoacceptors/isodecoders complicates both the analysis and read mapping. Existing databases for tRNA sequencing provide near exhaustive listings of tRNA genes, but the use of such highly redundant reference sequences in RNA-seq analyses leads to a large number of ambiguously mapped sequencing reads. Here we describe a relatively simple computational strategy for semi-automatic collapsing of highly redundant tRNA datasets into a non-redundant collection of reference tRNA sequences. The relevance of the approach was validated by analysis of experimentally obtained tRNA-sequencing datasets for different prokaryotic and eukaryotic model organisms. The data demonstrate that non-redundant tRNA reference sequences allow improving unambiguous mapping of deep sequencing data.

## 1. Introduction

Transfer RNA molecules (tRNAs) are essential adaptors in mRNA decoding, whereby these small ncRNA species are in charge for implementation of the genetic code at the molecular level [1,2]. These RNAs are heavily modified during post-transcriptional maturation steps [3,4] and, in consequence, analysis of these RNA species is of particular interest both for studies of mRNA translation [5,6], and for analysis of their epitranscriptomic modifications [7,8,9]. tRNAs are relatively abundant in the cell and represent ~10–15% of the total RNA content, depending on the organism [10]. Due to the presence of multiple species of similar size, and even similar sequence, isolation of individual tRNA species for analysis is possible, but relatively laborious. The most efficient methods are based on specific hybridization of complementary biotinylated DNA oligonucleotide, followed by affinity separation and specific elution [11,12]. Alternative methods of tRNA analysis, such as microarrays or microscale thermophoresis, give excellent results in quantification of different species in a pool [13,14], but are not high throughput techniques. Taking all of these considerations into account, the use of deep sequencing has become the most popular way for analysis of tRNA species, their relative abundance, and also their modification profiles. 

Analysis of tRNAs by deep sequencing is rather straightforward and may be performed directly from total RNA. For better efficiency, the tRNA population may be fractioned either by size selection (on agarose or polyacrylamide gels) or by selective extraction from cells [12]. Depletion of rRNA by Ribo-Zero^TM^ or equivalent “subtractive” methods to obtain tRNA enriched fraction can be used as well [12]. Library preparation is performed either by direct ligation of adaptors [15,16] to 5’- and 3’-ends of tRNA (such ligation protocol is also implemented in the majority of small RNA kits for library preparation) [8], or by primer extension using 3′-end ligated tRNA and tagging of the resulting cDNA [17,18,19]. In some protocols, preliminary fragmentation of tRNAs is used for analysis (e.g., in Hydro-tRNASeq and RiboMethSeq protocols) [20,21,22]. 

Whatever protocol is chosen for library preparation from tRNA fractions, the resulting sequencing reads have to be processed by alignment to the appropriate reference sequence, to allow meaningful biological interpretation. Thus, a major challenge in tRNA sequencing consists in construction of such precise and unambiguous alignment for further counting of tRNA reads and/or analysis of their post-transcriptional modifications. 

Multiple approaches were proposed as alignment strategies for tRNAs. Existing tRNA-oriented databases (the most popular are gtRNAdb [23] and tRNAdb-CE 2011 [24]), collect already published information and also propose prediction of tRNA genes in multiple species, generally using tRNAScan-SE [25,26]. These sources of information are extremely helpful, but, as a reference sequence, they propose either full genomic datasets of all tRNA genes or non-redundant collection of all possible tRNA species found (and predicted) in the genomic DNA. These reference sequences are certainly complete, but their direct use in bioinformatic analyses leads to numerous ambiguously (multiply) aligned reads. On the other hand, databases collecting experimental data on already sequenced tRNAs (MODOMICS [27], tRNAdb [28] and also T-psi-C database [29]) propose only validated sequences of existing and presumably most abundant tRNA species, however, for many reference/model organisms this information is scarce or simply not available (e.g., *Arabidopsis thaliana* or *Deinococcus radiodurans*). 

These limitations have so far prevented a generalized approach or common reference sequence for routine tRNA analysis by deep sequencing. Published analytical pipelines either use full genomic collection of tRNA species [7,8] (or better, non-redundant collections where identical species are already collapsed) [6,9] or apply rather laborious and multistep analytical pipelines involving alignment to full genomic DNA and extraction of mapped tRNA reads by coordinates, with subsequent collapsing of identical sequences at that stage [15,16,22,30,31]. These approaches give excellent results, but have been applied only to limited number of living species. Moreover, such complex multistep pipelines are not truly compatible with one another, precluding a direct comparison of the reported results. Furthermore, they are not suitable for routine analysis of tRNA pools in biomedical projects or for analysis of tRNA modification profiles when multiple species are involved. 

We propose a simple and reproducible algorithm as a tool for semi-automatic analysis of tRNA datasets starting from full and redundant genomic references identified by tRNA-ScanSE. The dataset, downloaded as fasta file (*.fa) from gtRNAdb or tRNAdb-CE 2011 was first collapsed in full automatic mode, to obtain a non-redundant tRNA reference. This reference may be used to extract all tRNA-related reads from experimental tRNA sequencing data. Full non-redundant tRNA reference was further analyzed in a semi-automatic way, to collapse closely related tRNA sequences in a single tRNA reference. The distance for collapsing into a unique entry is proposed by the algorithm and modified or validated by the user after visual inspection of the pairwise Levenshtein distance heatmap. These optimized non-redundant reference sequences were validated using experimental data available for several model living species and can be employed for routine analysis of tRNAs and their modifications. 

## 2. Materials and Methods 

### 2.1. Library Preparations

All deep sequencing tRNA libraries were prepared using the same protocol, implemented in the RiboMethSeq procedure [20,32]. Total RNA (or size-selected tRNA fraction) was subjected to fragmentation under strong alkaline conditions (10–15 min in 50 mM bicarbonate buffer pH 9.2 at 96 °C). Fragments were de-phosphorylated at the 3′-end and re-phosphorylated at the 5′-end to insure their compatibility with a direct adapter ligation step. Further steps were performed using the NEBNext Small RNA kit (E7330L) following the manufacturer’s recommendations. Quality of the libraries was assessed by HS DNA chip (Bioanalyzer 2100 Agilent). Sequencing of 50 nucleotides of the insert was performed in single-read SR50 mode on HiSeq 1000 Illumina sequencer. Target number of raw reads was 10–25 million, depending on the source of RNA (see Supplementary Table S1 for the exact number of sequencing reads used for analysis). Both total RNA and enriched tRNA fractions can be used for tRNA analysis, however, sequencing of total RNA should be done at much higher depth, to obtain sufficient amount of reads for all tRNAs. Since tRNAs represent ~5–15% of total RNA, ~10 times higher sequencing coverage is generally required in such cases. 

### 2.2. Computations

#### 2.2.1. tRNA Reference Sequence

Analysis of tRNA reference sequences was performed in R-studio with R version 3.5.3 and R packages seqinr and msa. Initial tRNA sequences (mature reference sequences) were downloaded from gtRNAdb (http://gtrnadb.ucsc.edu/GtRNAdb2/index.html), intron-containing sequences were not included in the analysis. No modification of the reference (except U->T conversion to get standard DNA *.fasta file) was performed prior to downstream analysis. Conserved 3’-CCA tRNA sequence was not introduced at this stage since 3’-CCA can be already encoded in genomic sequences, such as in *Escherichia coli*, and thus included in the sequences from gtRNAdb. However, this is not the case for *Homo sapiens*, and many other eukaryotes. In most complex cases like for *Bacillus subtilis*, some tRNA genes encode 3’-CCA while the others do not. This prevents the automatic addition of 3’-CCA to the reference sequence and a manual verification of tRNA cloverleaf structure and alignment is mandatory. 

**Figure 1:**
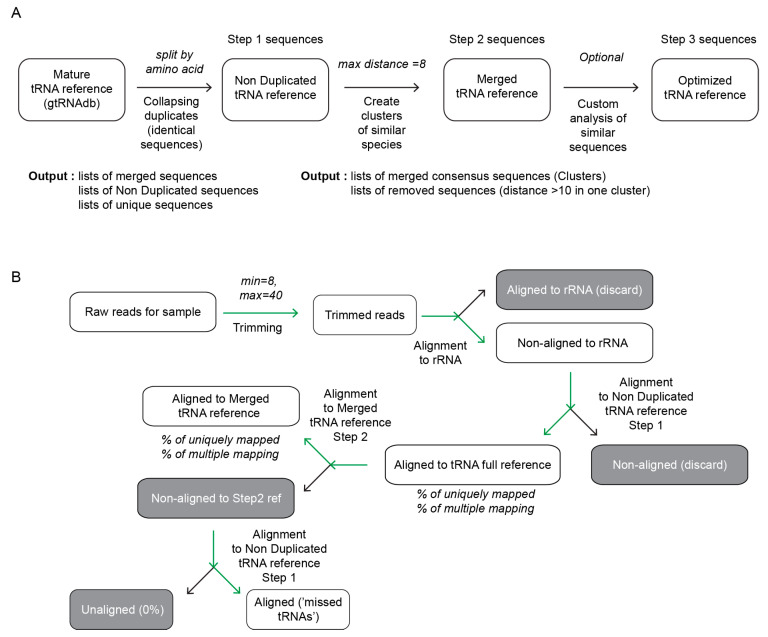
Strategy to create and validate a tRNA reference sequence. Panel (**A**)—collapsing of tRNAScan-SE predicted tRNA genes in non-duplicated (non-redundant) tRNA reference followed by further optimization (Step 2) by merging of closely related sequence (distance ≤ 8 by default). Further improvements may be introduced in a manual mode, by analysis of residual redundant mapping. Panel (**B**)—experimental analysis and validation of proposed tRNA reference sequences. Raw sequencing reads obtained in SR50 mode are trimmed and first filtered by alignment to rRNA reference sequence of the organism. Non-mapped reads are aligned to Step 1 (non-redundant) tRNA sequences. Mapped reads are re-aligned to Step 2, optimized reference and % of total and unique mapping are calculated. Finally, non-mapped Step 2 reads are re-analyzed by alignment to Step 1 reference, to get “missing tRNAs”.

The first step of analysis consists in the collapsing of identical sequences by a selection according to the amino-acid’s specificity, since “unexpected” anticodons (see definition in gtRNAdb) are relatively rare and eventual existence of such anticodon-mutated tRNA species is verified at the final treatment step. Identical sequences are identified and merged together (non-duplicated tRNA reference/Step1) in a fully automatic mode (see Figure 1A).

The second step aims to identify closely related tRNA species and collapse them into a unique consensus sequence, replacing ambiguous nucleotides by the nucleotide found in majority of sequences or by N, if the number is equivalent. We decided not to introduce International Union of Pure and Applied Chemistry (IUPAC) codes for ambiguous DNA residues since these codes are not systematically interpreted by popular alignment algorithms (such as Bowtie/Bowtie2 [33] used in this work). Collapsing into clusters is performed in semi-automatic mode, and a heatmap showing calculated Levenshtein distances between sequences is displayed to help the user to define the appropriate number of clusters. The script suggests the optimal number of distant clusters (with default max distance of 8 substitutions), but, if required, this number can be overridden by manual entry of a more appropriate value. Such manual correction is optional for the majority of simple bacterial and lower eukaryote tRNA references, but was found to be necessary for very complex clusters found for tRNA genes in higher eukaryotes. If the number of clusters to create is erroneously selected too low, the tRNA sequences, forced to merge in the same cluster, but still distant more than 10 substitutions are removed and stored in the “Removed sequence” file. 

After collapsing to clusters, a final heatmap is created for visual inspection and verification, sequences are re-annotated to use only the amino acid identity and anticodon sequence, isoacceptors/isodecoders with the same anticodon are numbered sequentially. 

Calculation scripts were tested for 20 organisms listed in the Table 1, Step 1 for merging identical sequences worked in all tested cases, Step 2 failed only when the number of fully identical sequences was > ~100, due to the excessive length of fasta file header, not accepted by R package seqinr. 

When the semi-automatic clustering of tRNA sequences was not sufficient to obtain <10–15% of multiply mapped reads after analysis of real tRNA datasets, tRNA reference was manually inspected and redundant isoforms creating ambiguous mapping were removed or collapsed in a single entry.

Finally, if not present in the genomic tRNA sequences, 3’-CCA end was manually added to the reference, to improve alignment quality in an end-to-end mode with sequencing reads issued from mature CCA-containing tRNA species. If present, anticodons with A34NN sequence were replaced by G34NN since A->I conversion at position 34 creates the residue behaving as G in base pairing.

#### 2.2.2. Alignment of the Experimental Datasets to Reference tRNA Sequences

Available experimental datasets obtained for total RNA or enriched tRNA fractions were treated by the same analytical pipeline. Demultiplexing and trimming was done without option for removal of potential PCR duplicates, since this is not appropriate for large datasets generated from relatively short reference sequence (here, collection of tRNAs). Raw reads were trimmed using Trimmomatic version 0.32 [34], using the following parameters (minLen 8 nt, maxLen 50 nt, single-end mode, stringency 7 (with these parameters the sequence of adapter over 10 nt is detected and removed). Taking into account very short cumulated reference sequence (maximum 250 sequences of 75–90 nt only, so <15 kb) minLen of 8 nt is, in principle, sufficient for unambiguous mapping. However, in practical, a proportion of very short reads may be still ambiguously aligned. Only reads <40 nt were selected for analysis to make sure that used raw reads are not contaminated by residual adapter sequence. Alignment of short reads was first performed to complete rRNAs sequence of the organism, since rRNAs are always dominating RNA species in total RNA and, very frequently, even in presumably ‘enriched’ tRNA fraction. Alignment was done by Bowtie2 v.2.2.4 [33], in sensitive (-D15-R2-N 0-L10-i S,1,1.15) end-to-end mode (no soft-trimming). Reads, non-aligned to rRNA sequences, were retained for further analysis, aligned to the non-duplicated tRNA reference/Step 1 sequences and all aligned reads were conserved (“tRNA-mapped reads”). Unique and multiply mapped reads for each tRNA were counted. The second tRNA alignment of all “tRNA-mapped reads” was done with an optimized tRNA reference (Step2 sequences), with the same Bowtie2 parameters. Unique and multiply mapped reads by tRNA were counted. Reads, non-aligned to optimized reference sequence, were conserved and re-aligned to non-duplicated tRNA reference/Step 1 sequences to identify tRNA species ‘missing’ in the optimized reference. 

### 2.3. Practical Guidelines for Optimization of the Reference tRNA Sequences

For practical analysis of tRNA dataset for an organism, we suggest the following pipeline:

1. Collapse full genomic tRNA dataset in collection of non-redundant sequences (automatic mode, Step 1).
2. Verify the number of non-duplicated sequences (Step 1), numbers of <60 indicate almost non-redundant dataset, higher values are indication of ambiguous redundant sequences.
3. Use the distance of 8 nt for genomic datasets of <250 tRNA genes (<60 Step 1 sequences) and the distance of 10 nt for larger genomic references. There may be intermediate cases for organisms having between 250 and 300 tRNA genes.
4. Verify the number of optimized (Step2) sequences, values close to 40 (or less) are indication of a good quality non-redundant reference, while numbers > ~50 mean still complex and potentially redundant tRNA collection.
5. Validate the optimized (Step 2) reference with experimentally obtained tRNA dataset. If proportion of uniquely mapped reads is still <90%, repeat collapsing in Step 2 with increased distance threshold.

## 3. Results

### 3.1. A Two-Steps Algorithm for tRNA Analysis

Depending on the complexity of the organism and the level of duplication, a full genomic tRNA reference sequence may be relatively simple (<100 tRNA genes in many bacteria and archaebacteria), of intermediate complexity (~120–250 tRNA genes in many bacteria and lower eukaryotes), or extremely complex with over 550 tRNA genes in *H. sapiens*/*Mus musculus*, *A. thaliana* and other higher eukaryotes (Table 1 and Supplementary Figure S1) [35]. This increased complexity generally results from duplication of the existing tRNA genes occasionally featuring point mutations in non-essential regions and not from the appearance of new and thus very different tRNA species [14,36]. In simple and intermediate cases, elimination (or collapsing them into one sequence) of redundant (fully identical) tRNA species is generally sufficient to obtain appropriate tRNA reference and further semi-automatic step to find and merge tRNA isodecoders with a limited number of point mutations is rather straightforward (Figure 1). 

### 3.2. Analysis of Simple tRNA References (<100 tRNA Genes)

For common bacterial species, the proposed approach was validated using tRNA references for *E. coli* and *B. subtilis*. The full genomic *E. coli* tRNA reference contains 89 tRNA genes (source gtRNAdb). Fully identical duplicated genes were found and collapsed, creating a non-redundant tRNA dataset of 48 tRNA sequences (“Step1”, see Table 1). Further collapsing of closely related tRNA species with the maximal distance of 8 substitutions gave an optimized tRNA reference of 41 tRNA genes. The representativeness of tRNA anticodons was verified and compared to the initial full dataset. All anticodons were found to be correctly represented, except for tRNA^Ala^ (GGC) and tRNA^Ala^ (TGC) which were merged in a single entry tRNA^Ala^ (NGC) with 7 substitutions and two tRNA^Thr^ (CGT) isodecoders, which were maintained, since relatively distant (see Supplementary Data). The optimized reference sequence for *E. coli* contains all tRNA species reported in MODOMICS and tRNAdb and is similar to the one used previously for tRNA modification analysis [21].

Similarly, collapsing of the full tRNA gene set from *B. subtilis* (86 sequences) gave 43 non-redundant tRNA sequences at the first step and 35 optimized tRNA references with a maximum of 8 allowed substitutions. The resulting optimized reference is fully representative for all used codons and adds 11 additional tRNA isoacceptors compared to MODOMICS/tRNAdb reference databases. 

Additional tests with other bacterial species demonstrated that semi-automatic collapsing is sufficient to obtain highly optimized tRNA references in such cases (see Table 1). The minimal number of retained tRNA genes is 31 (which is close to anticipated minimum required for correct mRNA decoding), and the average is about 40 species in low complexity genomic reference (<100 tRNA genes).

### 3.3. tRNA References of Intermediate Complexity (<300 Genes)

Collections of tRNA gene reference sequences of intermediate complexity are found in lower eukaryotes or representative insect species, such as *Saccharomyces cerevisiae* (275 tRNA genes in full genomic reference) or *Drosophila melanogaster* (total of 290 tRNA genes). For *S. cerevisiae*, an automatic merging of identical tRNAs reduced the number of tRNA sequences to 54 species and further optimization by collapsing of sequences with minimal distance of eight substitutions reduced this number to 38. Three closely related tRNA pairs showing very short distance (3–4 substitutions only, tRNA^Ser^ (CGA/TGA), tRNA^Glu^ (CTC/TTC) and tRNA^Gln^ (CTG/TTG)) were merged together, the other codons are represented as in the full genomic tRNA dataset. All sequences listed in MODOMICS/tRNAdb are present in the optimized reference sequence, but this last reference contains 10 supplementary tRNA species compared to MODOMICS /tRNAdb. 

For the *D. melanogaster* reference (290 tRNA genes), collapsing of fully identical sequences gave 76 tRNA genes and further optimization reduced this number to 37 representative sequences. This list will be further reduced to 34 (see Section 3.5 and Supplementary Material, Section “Detailed description of modifications in final tRNA references” for more details). All sequences listed in MODOMICS/tRNAdb are also present in this reference and it contains 20 additional tRNA sequences.

Analysis of other genomes from this group showed that the minimal number of tRNA genes retained in optimized references is close to 35, while the maximum increases to about 60, indicating considerable diversity (see Table 1). Noteworthy, the numbers of tRNA species in non-redundant reference and in the optimized one are not directly proportional to a total number of tRNA genes in the genome.

### 3.4. Highly Complex tRNA References (>400 Genes)

Higher eukaryotic genomes contain substantial numbers of tRNA genes (see Table 1), mostly due to duplicated sequences and multiple point mutants. Depending on the genome build, human tRNA reference sequence lists >400 tRNA genes, even more tRNA sequences were found in the *C. elegans* worm genome (568 in “high confidence set”, gtRNAdb) and the *A. thaliana* plant genome has 580 sequences in “high confidence set”. Even after collapsing of identical tRNA sequences, the residual non-redundant tRNA reference remains very large (>150 non-identical tRNA genes). However, these genes form large clusters of related sequences. Here we analyzed *H. sapiens* and *A. thaliana* tRNA genes in more detail, since human tRNAs were in a large proportion directly sequenced in the past and included in MODOMICS and tRNAdb, while only very limited information exists on the tRNAs from *A. thaliana*. The human non-redundant tRNA reference has 177 non-identical tRNA genes, while collapsing with the default 8 nt distance gave 61 entries in Step2 Optimized sequences. Despite a larger genomic tRNA gene collection (590 sequences), *A. thaliana* tRNA genes were found to be less diverse, leading to only 133 entries in non-redundant Step1 collection and 48 after Step2 (see Table 1).

The algorithm was also tested for highly complex models, like plant *Z. mais* (770 tRNA genes) and frog *X. tropicalis*, where >3000 tRNA genes were found. Collapsed reference contains, respectively, 191 and 245 non-redundant tRNA species, while “optimized dataset” uses only 70 and 68 representative tRNA sequences (Table 1).

### 3.5. Validation of the Optimized tRNA Reference Sequences

For an experimental validation we selected six representative tRNA reference sequences: two from bacterial, common model organisms *E. coli* and *B. subtilis*, respectively, from Gram− and Gram+ groups, and four from eukaryotic species showing rather variable number of tRNA genes in the genome and optimized tRNA references: *S. cerevisiae*, *D. melanogaster*, *H. sapiens* and *A. thaliana* (see Table 1, in red). For each organism, RiboMethSeq-like datasets were prepared for 6–20 samples, from total RNA, or from size-selected tRNA fraction. Alignment statistics for analyzed datasets is shown in Table S1. 

Raw trimmed reads were cleaned up from rRNA contamination and aligned first to non-duplicated (non-redundant) full tRNA reference (Step 1, see Figure 1B). Mapped reads were extracted and used for further analysis by alignment to an optimized tRNA reference (Step 2). In both cases uniquely and multiply mapped reads were counted, globally, as well as by individual tRNA. 

**Figure 2:**
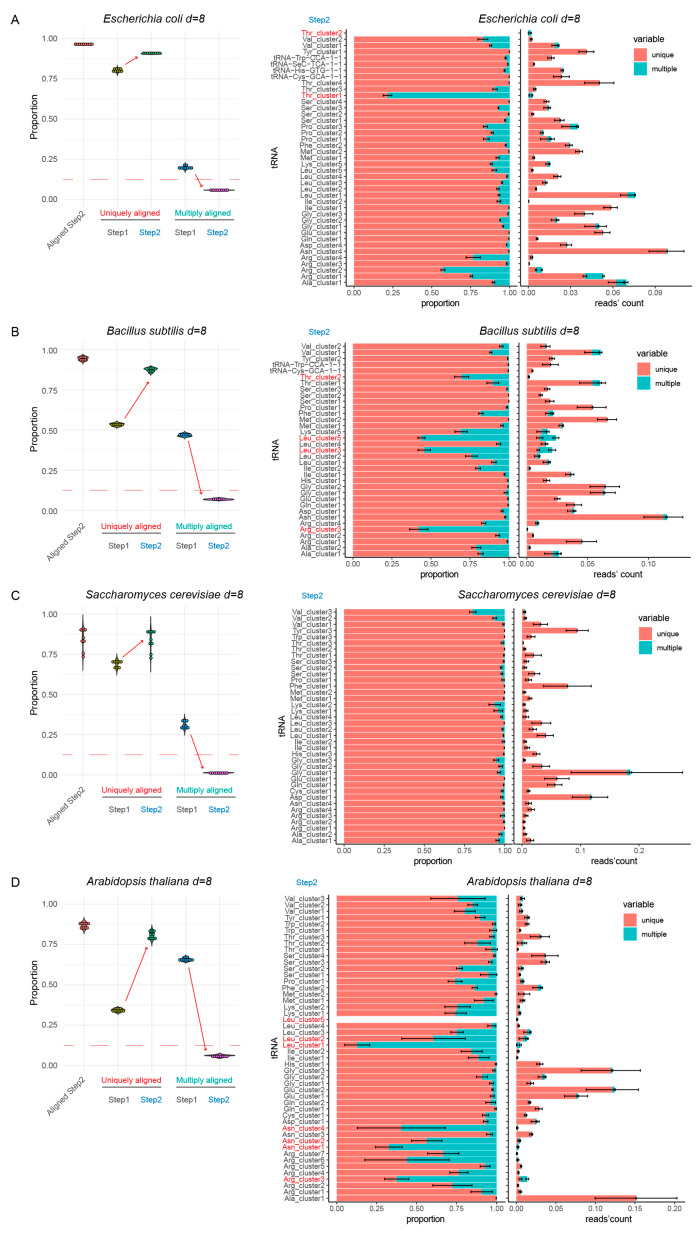
Alignment results for non-duplicated (non-redundant) tRNA reference (Step 1) and optimized tRNA set (Step 2) for four model species, with a maximum distance of eight substitutions. Panels (**A**–**D**) show analysis of *Escherichia coli, Bacillus subtilis, Saccharomyces cerevisiae* and *Arabidopsis thaliana* tRNA references, respectively. Boxplot on the left shows the proportion of tRNA sequencing reads aligned to Step 2 reference (“Aligned Step 2”) and proportions of uniquely and multiply mapped reads at both steps. Red dashed lines indicate the 12.5% level. The increase of unique mapping events and the decrease of multiple mapping events are shown by arrows. Barplots at the right represent unique and multiple mapping by tRNA species at Step 2, in proportion to total and in absolute number of sequencing reads obtained by tRNA, expressed as proportion to total number of mapped reads. tRNAs showing excessive proportion of ambiguous mapping are shown in red.

The results shown in Figure 2 (and Supplementary Figure S2) demonstrated that for all species tested, >92–95% of total tRNA reads were still aligned to optimized reference (“Aligned Step 2”), showing that collapsing to clusters did not reduce alignment quality. However, the proportion of the uniquely mapped reads increased very drastically with Step 2 reference sequences, with concomitant decrease of ambiguous multiple mapping (Figure 2, compare uniquely and multiply aligned for Step 1 and Step 2). In the best cases, the residual multiple mapping was <5%, and routinely <10% for simple tRNA references. Only minor further adaptations were necessary to remove/manually collapse residual sequences with ambiguous mapping from *E. coli, B. subtilis,* and *A. thaliana* references (indicated in red on Figure 2 and described in Supplement, Section “Detailed description of modifications in final tRNA references”).

**Figure 3:**
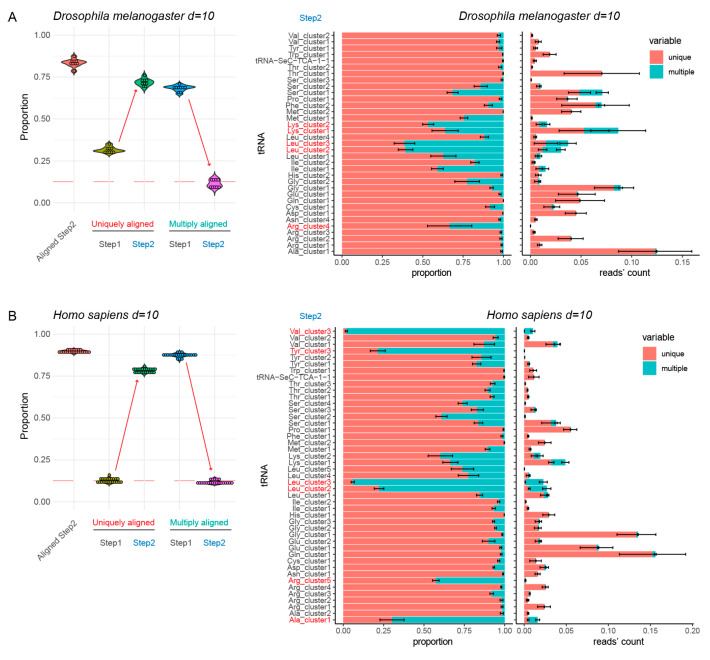
Alignment results for non-duplicated (non-redundant) tRNA reference (Step1) and optimized tRNA set (Step 2) for two complex model species, maximal distance used is 10 substitutions. Panels (**A**,**B**) show analysis of *Drosophila melanogaster* and *Homo sapiens* tRNA references, respectively. Boxplot on the left shows the proportion of tRNA sequencing reads aligned to Step2 reference (“Aligned Step 2”) and proportions of uniquely and multiply mapped reads at both steps. Red dashed line indicates 12.5% level. Increase of unique mapping and decrease of multiple mapping is shown by arrows. Barplots at the right represent unique and multiple mapping by tRNA species at Step2, in proportion to total and in absolute number of sequencing reads obtained by tRNA, expressed as proportion to total number of mapped reads. tRNAs showing excessive proportion of ambiguous mapping are shown in red.

However, when the standard distance of eight substitutions was applied to human and drosophila tRNA references, residual ambiguous mapping was still quite substantial (>15%) (see Supplementary Figure S2). This is certainly related to the excessive number of rather similar but non-merged tRNA sequences. Thus, we explored a semi-automatic fusion with an increased number of allowed substitutions (up to 10). Manual correction of the suggested cluster number was systematically required for such analysis. The final reference for *H. sapiens* contained only 45 sequences, instead of 61 for a maximal distance of eight substitutions (see Table 1). Similarly, the drosophila tRNA reference sequence was reduced to 35 unique entries. Analysis of uniquely and multiply mapped reads from experimental datasets showed substantial improvement compared to the previous reference sequence with *n* = 8 as maximum distance. The proportion of multiply mapped reads decreased to ~10–12%, which was considered acceptable (see Figure 3).

Inspection of multiply mapped reads showed that only tRNA^Leu^, tRNA^Lys^ and tRNA^Arg^ in the *D. melanogaster* dataset still showed substantial multiple mapping (shown in red, Figure 3). For the human reference, this was the case for pairs of tRNA^Leu^, tRNA^Arg^, one tRNA^Tyr^ and tRNA^Ala^/tRNA^Val^ (Figure 3). These cases were manually inspected to find the origin of such ambiguous mapping. Both in *D. melanogaster* and in human, two tRNA^Leu^ isoacceptors were found well represented in the RNA-seq data and showed very similar sequences at the 5′- and 3′-extremities but differed in the anticodon loop (Supplementary Figure S3 and information in the Supplementary Data). Human tRNA^Tyr^_3_ reads were rather scarce compared to two other tRNA^Tyr^ isoacceptors and thus tRNA^Tyr^_3_ was removed from the curated reference sequence. Finally, tRNA^Val^_3_ (encoded by the unique gene tRNA-Val-AAC-6-1) differs from tRNA^Ala^_1_ only at 10 positions and appeared to be an anticodon (missense suppressor) mutant of tRNA^Ala^_1_. Thus, this tRNA^Val^_3_ was also removed from the final reference sequence list.

Final manually curated reference sequences for six model organisms now include the 3′-CCA end, and all anticodon A34 residues were replaced by G since A34 is systematically converted to inosine (I) [37], which behaves as G in base pairing. Alignment results of full tRNA reads to curated references are shown in Table 2.

## 4. Discussion

### 4.1. Merging of Similar tRNA Genes in a Single Reference Sequence

Highly redundant RNA sequences issued from gene duplication and sequence mutations are difficult targets for extensive and unambiguous bioinformatic analysis of deep sequencing data, since short sequencing reads obtained in many popular protocols do not allow to distinguish between these very similar species. Transfer RNA (tRNA) populations in most organisms are well-studied examples. Most bacterial species have 60–80 tRNA encoding genes, and this number increases rapidly with genome complexity. Most complex (from a tRNA gene-centered point of view) eukaryotic species may contain >5000 duplicated and point-mutated tRNA genes. After removal of duplicates, over 100 (or even more) distinct sequences are still present. Analysis of such extremely complex mixtures requires unambiguous mapping, which is only possible with long sequencing reads. Only reads over 30–40 nt in length are truly unique enough to be precisely mapped to a given RNA sequence. However, such reads are relatively rare in the case of tRNA isoacceptors/isodecoders, which are of 75–90 nt in length only and heavily modified, preventing, in most cases, trouble-free read-through by reverse transcriptase (RT). tRNA-demethylation protocols (e.g., demethylase tRNA sequencing, DM-tRNA-Seq, AlkB-facilitated RNA methylation sequencing, ARM-Seq, etc.) improve efficiency of primer extension, but do not remove all RT-blocking modified nucleotides. Thus, tRNA deep sequencing data are known to by heavily biased, with preferential amplification of only certain tRNA species [13]. Up to now, no general approach allowing rapid analysis of tRNA deep sequencing data was established in the community, and so far, every new organism required particular attention and almost manual inspection of numerous tRNA sequences. 

In this work, we propose a generalized approach to reduce the complexity of tRNA reference sequences, intended to be used for reads mapping. The first, most evident, step consists in removal of all duplicated tRNA genes of exactly identical sequence. This step is efficient and allows reducing complexity very considerably. However, such automatic removal of duplicates does not provide unambiguous mapping reference, since point-mutated variants persist. Such non-redundant reference is suitable for mapping of long (preferentially full size) tRNA reads, obtained without RNA fragmentation [15,16]. Unfortunately, such non-fragmented tRNA libraries are highly biased due to certain tRNA modifications resulting in abortive (incomplete) cDNA fragments. Alternative approaches using tRNA fragmentation are more efficient, since fragments may be devoid of RT-arresting modifications; however, shorter sequencing reads are obtained in such cases [22]. This is also a feature of the RiboMethSeq protocol, which is now extensively used for analysis of tRNA 2′-O-methylations [20,21]. Other protocols, such as AlkAnilineSeq for detection of m^7^G/m^3^C/D/ho^5^C [38] and HydraPsiSeq for pseudouridine mapping and quantification [39], also result in relatively short tRNA reads.

To obtain unambiguous reads mapping, which may ultimately be used for both tRNA quantification and modification analysis, we propose to group (merge) closely related species into a single entry, replacing “ambiguous” mutated nucleotides by most frequent nucleotide or by N. Even if the use of IUPAC nucleotide code seems to be more appropriate, such nucleotides are considered as N by many alignment utilities (Bowtie2, STAR, BWA) [40,41]. The number of N nucleotides should be limited, to reduce ambiguous mapping, but not become too small, since otherwise closely related species will lead to multiple mapping events. Empirically, *N* = 7–8 maximum (10% of the tRNA length) was found to be suitable for the majority of simple tRNA references (bacteria/archaea/lower eukaryotes), while 10–11 substitutions have to be allowed for more complex cases, such as human and drosophila tRNAomes. 

### 4.2. Representativeness of Optimized tRNA Datasets

In order to check if such reduced tRNA reference datasets remain representative, anticodon compositions of full and optimized datasets were compared (see Supplementary Materials). In general, when some apparent loss of rarely used tRNA anticodons was found, manual inspection revealed that those correspond to rare tRNA isoacceptors (encoded by genes with low copy number) and represent point mutant of major tRNA isoacceptor. These conversions are generally limited to tRNAs decoding the same amino acid, with an exception of one human tRNA^Val^ (AAC), which is in fact derived from tRNA^Ala^ (NGC) by a limited number of mutations, including two in the anticodon. Interestingly, this tRNA^Val^ (AAC) is most likely behaving as a missense suppressor in vivo since it retains a G3*U70 base pair and a discriminator nucleotide A73, two well-known identity determinants for aminoacylation by alanine tRNA-synthetase [42].

Analysis of several experimentally available tRNA datasets showed that only minor fractions (<10%) in these whole collections of tRNA reads were not aligned to the optimized reference sequence (Supplementary Figure S4) and, thus, the representativeness of the reduced reference was clearly maintained.

### 4.3. Known Limitations and Troubleshooting

Counting of tRNA reads and comparative studies of tRNA expression are possible with a reduced/optimized reference; however, if only one particular tRNA isoacceptor has to be quantified, the best approach would be to use a non-redundant tRNA dataset and to select only (rare) uniquely mapped reads for a given tRNA. Analysis of tRNA modification with a reduced dataset is based on the assumption that all species in the group have identical tRNA modification profiles, independently from the presence of a point mutation. This assumption is generally relevant, since mutations occur mainly in the tRNA regions, which are not known to be extensively modified; however, this may not be true for some essential tRNA anticodon positions (N34, for example). In such cases, inspection of nucleotide proportion (e.g., by Integrative Genomics Viewer (IGV) visualization or mismatch calculation from mpileup format) may be of interest to determine the proportion of really expressed tRNA variants. In extreme cases, preliminary evaluation of tRNA expression using non-redundant datasets may be required, followed by the analysis of only major expressed tRNA isoforms. However, this complex two-step methodology cannot be applied in an automatized routine analysis. 

## 5. Conclusions

### Applications in Analysis of tRNA Expression and Modifications

Since currently used approaches for tRNA analysis by deep sequencing suffer from multiple and thus very ambiguous mapping of tRNA reads, we foresee multiple practical applications of the proposed general approach for tRNA reference sequences. Unified and straightforward analysis of tRNA deep sequencing data is still lacking in the field. Quantification of tRNA pool and modulations of its composition under stress or in disease represent one of the major applications. One other major field that will certainly benefit from these improvements is analysis of epitranscriptomic tRNA modifications (2’-O-methylation, pseudouridine, m^7^G/m^3^C, D and others), important for adaptation of cellular translational machinery in stress or disease. With some minor adaptations, similar computational and validation approaches can be also applied for simplification of other highly redundant collection of RNA genes, e.g., rRNA and snRNA sequences. 

## Figures and Tables

**Table 1:** tRNA reference datasets for selected model organisms.

Organism	Domain	“High Confidence Set” gtRNAdb ^1^	Step1 Non-Redundant tRNA Set	Step2 Optimized “Collapsed” tRNA Set (d = 8)	Step3 Validated “Optimized” tRNA Set
*Plasmodium falciparum 3D7*	E	45	45	40	
*Sulfolobus acidocaldarius N8*	A	50	50	33	
*Haloferax volcanii DS2*	A	52	46	46	
*Staphylococcus aureus subsp. aureus NCTC 8325*	B	59	**43**	**31**	
*Leishmania major strain Friedlin (ASM272v2)*	E	82	49	44	
*Bacillus subtilis subsp. subtilis str. 168*	B	86	50	35	33
*Escherichia coli str. K-12 substr. MG1655*	B	89	48	41	39
*Candida albicans A20*	E	129	50	41	
*Candida albicans WO-1*	E	146	73	58	
*Schizosaccharomyces pombe 972h-*	E	171	60	44	
*Saccharomyces cerevisiae PW5*	E	171	47	42	
*Candida glabrata CBS 138*	E	189	45	38	
*Candida tropicalis 121*	E	203	70	57	
*Saccharomyces cerevisiae P301*	E	226	49	37	
*Saccharomyces cerevisiae S288c*	E	275	54	38	38
*Drosophila melanogaster (BDGP Rel. 6/dm6)*	E	290	76	37 35 (d = 10)	34
*Homo sapiens (GRCh37/hg19)*	E	416	177	61 45 (d = 10)	43
*Bombyx mori* (Domestic silkworm ASM15162v1)	E	435	115	44	
*Arabidopsis thaliana (TAIR10 Feb 2011)*	E	580	139	48	43
*Zea mays* B73 (RefGen_v4 AGPv4)		771	191	**70**	
*Strongylocentrotus purpuratus* (S. purpuratus) Mar. 2015 Spur_4.2)	E	931	192	61	
*Xenopus_tropicalis*_v9.1	E	3010	**245**	68	

^1^ Table is sorted in ascending order of predicted “high-confidence” tRNA genes (source gtRNAdb). Bolded and underlined values shows the minimal and maximal values.

**Table 2:** Alignment of full tRNA reads dataset to manually curated tRNA references (Step 3).

Organism	tRNA Gene Number	% of Aligned Reads	Uniquely Aligned Reads ^1^	Multiply Aligned Reads ^1^
*Escherichia_coli_str_K-12_substr_MG1655*	39	95.87 ± 0.26	95.95 ± 0.23	4.05 ± 0.23
*Bacillus_subtilis_subsp_subtilis_str_168*	33	94.89 ± 0.91	93.93 ± 0.38	6.07 ± 0.38
*Saccharomyces_cerevisiae_S288c*	38	84.93 ± 6.61	98.52 ± 0.27	1.48 ± 0.27
*Arabidopsis_thaliana_TAIR108feb2011*	43	85.93 ± 2.34	94.08 ± 0.93	5.92 ± 0.93
*Drosophila_melanogaster_BDGP6_dm6*	34	83.67 ± 2.36	86.88 ± 2.36	13.12 ± 2.36
*Homo_sapiens_GRCh37hg19*	43	90.08 ± 0.70	89.15 ± 0.92	10.85 ± 0.92

^1^ Normalized to the total number of aligned reads.

## Data Availability

All scripts, supplementary data, as well as non-redundant and optimized datasets are available from GitHub (https://github.com/FlorianPichot/tRNA_reference_construction).

## Supplementary Materials

The following are available online at https://www.mdpi.com/2073-4425/12/1/81/s1, Figure S1 Number of genomic tRNA genes detected by tRNAScanSE (source gtRNAdb). Genomes (~250 in total) were randomly chosen to provide representative selection for Archaea (35 genomes), Bacteria (150 genomes) and Eukaryota (65 genomes). Only tRNA genes corresponding to 20 standard amino acids were considered. Panel A represents global distribution (number of tRNA genes in log10 scale), panel B—same data sorted by Kingdom. Panel C shows the number of tRNA genes in non-redundant (blue) and in optimized (Step2) references (red), in function of the total number of tRNA genes in genomic reference. Panel D, E and F show distribution by Kingdom and phyla/groups. Figure S2: Alignment results for non-duplicated (non-redundant) tRNA reference (Step1) and optimized tRNA set (Step2) for *D. melanogaster* and *H. sapiens* references, maximal distance used is 8 substitutions, Figure S3: Sequences of tRNA species showing excessive ambiguous mapping for *D. melanogaster* and *H. sapiens* references, Figure S4: Barplots representing unique and multiple mapping by tRNA species for final manually curated tRNA references (Step3). Table S1: Characteristics of deep sequencing datasets used for analysis.

## References

[1] Berg M.D., Brandl C.J. (2020). Transfer RNAs: Diversity in form and function. RNA Biol..

[2] Lei L., Burton Z.F. (2020). Evolution of Life on Earth: tRNA, Aminoacyl-tRNA Synthetases and the Genetic Code. Life.

[3] Phizicky E.M., Hopper A.K. (2010). tRNA biology charges to the front. Genes Dev..

[4] Hori H. (2014). Methylated nucleosides in tRNA and tRNA methyltransferases. Front Genet.

[5] Wilusz J.E. (2015). Controlling translation via modulation of tRNA levels. Wiley Interdiscip. Rev. RNA.

[6] Pinkard O., McFarland S., Sweet T., Coller J. (2020). Quantitative tRNA-sequencing uncovers metazoan tissue-specific tRNA regulation. Nat. Commun..

[7] Clark W.C., Evans M.E., Dominissini D., Zheng G., Pan T. (2016). tRNA base methylation identification and quantification via high-throughput sequencing. RNA.

[8] Cozen A.E., Quartley E., Holmes A.D., Hrabeta-Robinson E., Phizicky E.M., Lowe T.M. (2015). ARM-seq: AlkB-facilitated RNA methylation sequencing reveals a complex landscape of modified tRNA fragments. Nat. Methods.

[9] Warren J.M., Salinas-Giegé T., Hummel G., Coots N.L., Svendsen J.M., Brown K.C., Drouard L., Sloan D.B. (2020). Combining tRNA sequencing methods to characterize plant tRNA expression and post-transcriptional modification. RNA Biol..

[10] Lodish H., Berk A., Zipursky S.L., Matsudaira P., Baltimore D., Darnell J. (2000). Processing of rRNA and tRNA. Molecular Cell Biology.

[11] Drino A., Oberbauer V., Troger C., Janisiw E., Anrather D., Hartl M., Kaiser S., Kellner S., Schaefer M.R. (2020). Production and purification of endogenously modified tRNA-derived small RNAs. RNA Biol..

[12] Kanwal F., Lu C. (2019). A review on native and denaturing purification methods for non-coding RNA (ncRNA). J Chromatogr B Analyt Technol. Biomed. Life Sci..

[13] Jacob D., Thüring K., Galliot A., Marchand V., Galvanin A., Ciftci A., Scharmann K., Stock M., Roignant J.-Y., Leidel S.A. (2019). Absolute quantification of noncoding RNA by microscale thermophoresis. Angew. Chem. Int. Ed. Engl..

[14] Coughlin D.J., Babak T., Nihranz C., Hughes T.R., Engelke D.R. (2009). Prediction and verification of mouse tRNA gene families. RNA Biol..

[15] Shigematsu M., Honda S., Loher P., Telonis A.G., Rigoutsos I., Kirino Y. (2017). YAMAT-seq: An efficient method for high-throughput sequencing of mature transfer RNAs. Nucleic Acids Res..

[16] Erber L., Hoffmann A., Fallmann J., Betat H., Stadler P.F., Mörl M. (2020). LOTTE-seq (Long hairpin oligonucleotide based tRNA high-throughput sequencing): Specific selection of tRNAs with 3’-CCA end for high-throughput sequencing. RNA Biol..

[17] Hauenschild R., Tserovski L., Schmid K., Thüring K., Winz M.-L., Sharma S., Entian K.-D., Wacheul L., Lafontaine D.L.J., Anderson J. (2015). The reverse transcription signature of N-1-methyladenosine in RNA-Seq is sequence dependent. Nucleic Acids Res..

[18] Tserovski L., Marchand V., Hauenschild R., Blanloeil-Oillo F., Helm M., Motorin Y. (2016). High-throughput sequencing for 1-methyladenosine (m(1)A) mapping in RNA. Methods.

[19] Zheng G., Qin Y., Clark W.C., Dai Q., Yi C., He C., Lambowitz A.M., Pan T. (2015). Efficient and quantitative high-throughput tRNA sequencing. Nat. Methods.

[20] Marchand V., Blanloeil-Oillo F., Helm M., Motorin Y. (2016). Illumina-based RiboMethSeq approach for mapping of 2’-O-Me residues in RNA. Nucleic Acids Res..

[21] Marchand V., Pichot F., Thüring K., Ayadi L., Freund I., Dalpke A., Helm M., Motorin Y. (2017). Next-Generation Sequencing-Based RiboMethSeq Protocol for Analysis of tRNA 2’-O-Methylation. Biomolecules.

[22] Gogakos T., Brown M., Garzia A., Meyer C., Hafner M., Tuschl T. (2017). Characterizing Expression and Processing of Precursor and Mature Human tRNAs by Hydro-tRNAseq and PAR-CLIP. Cell Rep..

[23] Chan P.P., Lowe T.M. (2016). GtRNAdb 2.0: An expanded database of transfer RNA genes identified in complete and draft genomes. Nucleic Acids Res..

[24] Abe T., Ikemura T., Sugahara J., Kanai A., Ohara Y., Uehara H., Kinouchi M., Kanaya S., Yamada Y., Muto A. (2011). tRNADB-CE 2011: tRNA gene database curated manually by experts. Nucleic Acids Res..

[25] Lowe T.M., Eddy S.R. (1997). tRNAscan-SE: A program for improved detection of transfer RNA genes in genomic sequence. Nucleic Acids Res..

[26] Zou Q., Guo J., Ju Y., Wu M., Zeng X., Hong Z. (2015). Improving tRNAscan-SE Annotation Results via Ensemble Classifiers. Mol. Inform..

[27] Boccaletto P., Machnicka M.A., Purta E., Piatkowski P., Baginski B., Wirecki T.K., de Crécy-Lagard V., Ross R., Limbach P.A., Kotter A. (2018). MODOMICS: A database of RNA modification pathways. 2017 update. Nucleic Acids Res..

[28] Jühling F., Mörl M., Hartmann R.K., Sprinzl M., Stadler P.F., Pütz J. (2009). tRNAdb 2009: Compilation of tRNA sequences and tRNA genes. Nucleic Acids Res..

[29] Sajek M.P., Woźniak T., Sprinzl M., Jaruzelska J., Barciszewski J. (2020). T-psi-C: User friendly database of tRNA sequences and structures. Nucleic Acids Res..

[30] Hoffmann A., Fallmann J., Vilardo E., Mörl M., Stadler P.F., Amman F. (2018). Accurate mapping of tRNA reads. Bioinformatics.

[31] Torres A.G., Reina O., Stephan-Otto Attolini C., Ribas de Pouplana L. (2019). Differential expression of human tRNA genes drives the abundance of tRNA-derived fragments. Proc. Natl. Acad. Sci. USA.

[32] Galvanin A., Ayadi L., Helm M., Motorin Y., Marchand V. (2019). Mapping and Quantification of tRNA 2’-O-Methylation by RiboMethSeq. Methods Mol. Biol..

[33] Langmead B., Salzberg S.L. (2012). Fast gapped-read alignment with Bowtie 2. Nat. Methods.

[34] Bolger A.M., Lohse M., Usadel B. (2014). Trimmomatic: A flexible trimmer for Illumina sequence data. Bioinformatics.

[35] Fujishima K., Kanai A. (2014). tRNA gene diversity in the three domains of life. Front. Genet..

[36] Wald N., Margalit H. (2014). Auxiliary tRNAs: Large-scale analysis of tRNA genes reveals patterns of tRNA repertoire dynamics. Nucleic Acids Res..

[37] Chen P., Qian Q., Zhang S., Isaksson L.A., Björk G.R. (2002). A cytosolic tRNA with an unmodified adenosine in the wobble position reads a codon ending with the non-complementary nucleoside cytidine. J. Mol. Biol..

[38] Marchand V., Ayadi L., Ernst F.G.M., Hertler J., Bourguignon-Igel V., Galvanin A., Kotter A., Helm M., Lafontaine D.L.J., Motorin Y. (2018). AlkAniline-Seq: Profiling of m7 G and m3 C RNA Modifications at Single Nucleotide Resolution. Angew. Chem. Int. Ed. Engl..

[39] Marchand V., Pichot F., Neybecker P., Ayadi L., Bourguignon-Igel V., Wacheul L., Lafontaine D.L.J., Pinzano A., Helm M., Motorin Y. (2020). HydraPsiSeq: A method for systematic and quantitative mapping of pseudouridines in RNA. Nucleic Acids Res..

[40] Li H., Durbin R. (2009). Fast and accurate short read alignment with Burrows-Wheeler transform. Bioinformatics.

[41] Dobin A., Davis C.A., Schlesinger F., Drenkow J., Zaleski C., Jha S., Batut P., Chaisson M., Gingeras T.R. (2013). STAR: Ultrafast universal RNA-seq aligner. Bioinformatics.

[42] Lovato M.A., Chihade J.W., Schimmel P. (2001). Translocation within the acceptor helix of a major tRNA identity determinant. EMBO J..

