# Supplementary material for: Non-Redundant tRNA Reference Sequences for Deep Sequencing Analysis of tRNA Abundance and Epitranscriptomic RNA Modifications
Source: Genes (Basel). 2021 Jan 10;12(1):81. doi: 10.3390/genes12010081 (PMC7827920; doi:10.3390/genes12010081)
Supplement: Supplementary file 1 [file genes-12-00081-s001.pdf]

Supplement

Non-redundant tRNA reference sequences for deep sequencing analysis of tRNA abundance and epitranscriptomics modifications

Florian PICHOT[1,2], Virginie MARCHAND[2], Mark HELM[1], Yuri MOTORIN[2,3*]

Figure S1 Number of genomic tRNA genes detected by tRNAScanSE (source gtRNAdb). Genomes (~250 in total) were randomly chosen to provide representative selection for Archaea (35 genomes), Bacteria (150 genomes) and Eukaryota (65 genomes). Only tRNA genes corresponding to 20 standard amino acids were considered. Panel A represents global distribution (number of tRNA genes in log10 scale), panel B – same data sorted by Kingdom. Panel C shows the number of tRNA genes in non-redundant (blue) and in optimized (Step2) references (red), in function of the total number of tRNA genes in genomic reference. Panel D, E and F show distribution by Kingdom and phyla/groups.

Figure S2: Alignment results for NonDuplicated (non-redundant) tRNA reference (Step1) and optimized tRNA set (Step2) for *D. melanogaster* and *H. sapiens* references, maximal distance used is 8 substitutions

Figure S3: Sequences of tRNA species showing excessive ambiguous mapping for *D. melanogaster* and *H. sapiens* references

Figure S4: Barplots representing unique and multiple mapping by tRNA species for final manually curated tRNA references (Step3).

Table S1: Characteristics of deep sequencing datasets used for analysis.

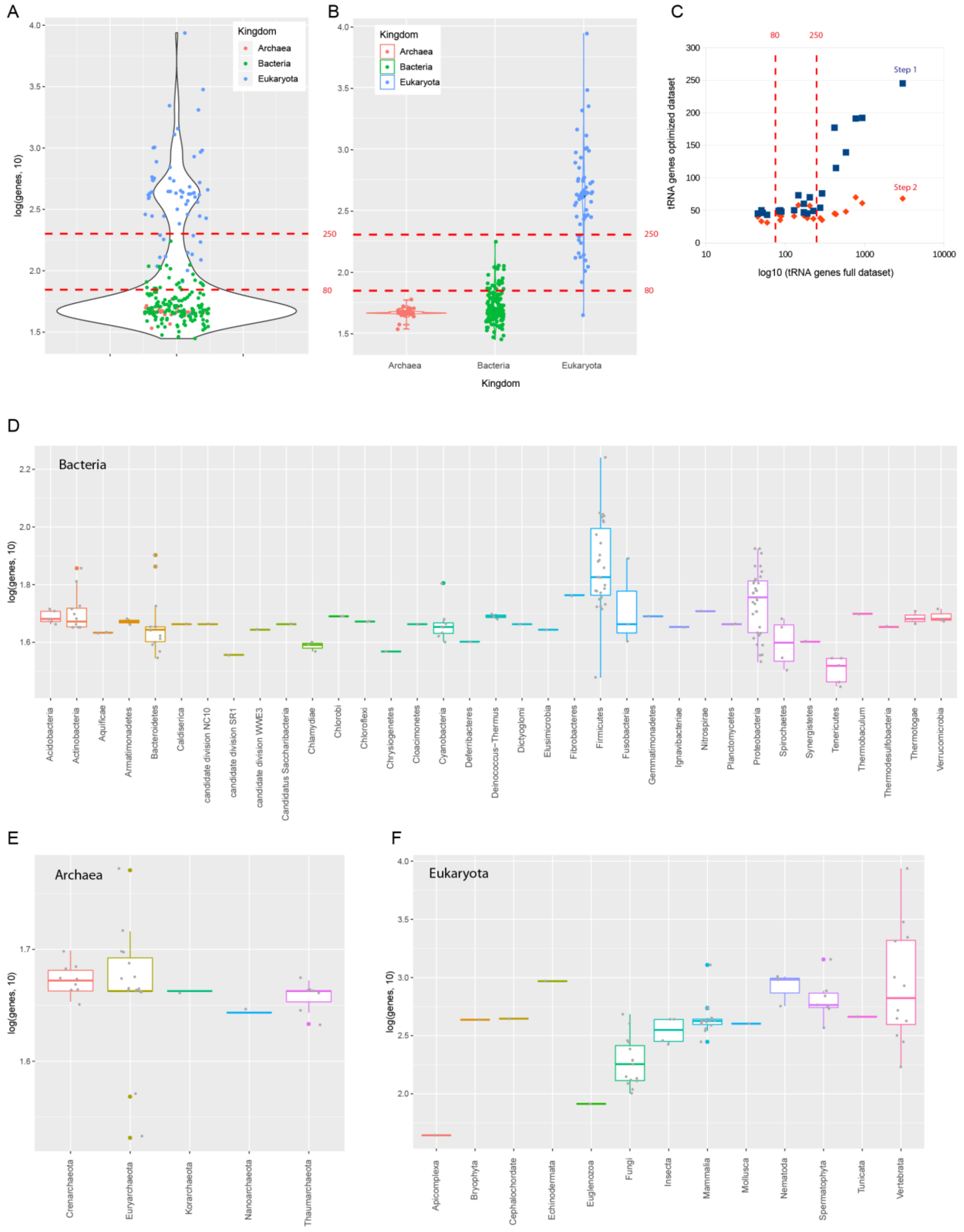

Figure S1 Number of genomic tRNA genes detected by tRNAScanSE (source gtRNAdb). Genomes (~250 in total) were randomly chosen to provide representative selection for Archaea (35 genomes), Bacteria (150 genomes) and Eukaryota (65 genomes). Only tRNA genes corresponding to 20 standard amino acids were considered. Panel A represents global distribution (number of tRNA genes in log_{10} scale), panel B – same data sorted by Kingdom. Panel C shows the number of tRNA genes in non-redundant (blue) and in optimized (Step2) references (red), in function of the total number of tRNA genes in genomic reference. Panel D, E and F show distribution by Kingdom and phyla/groups.

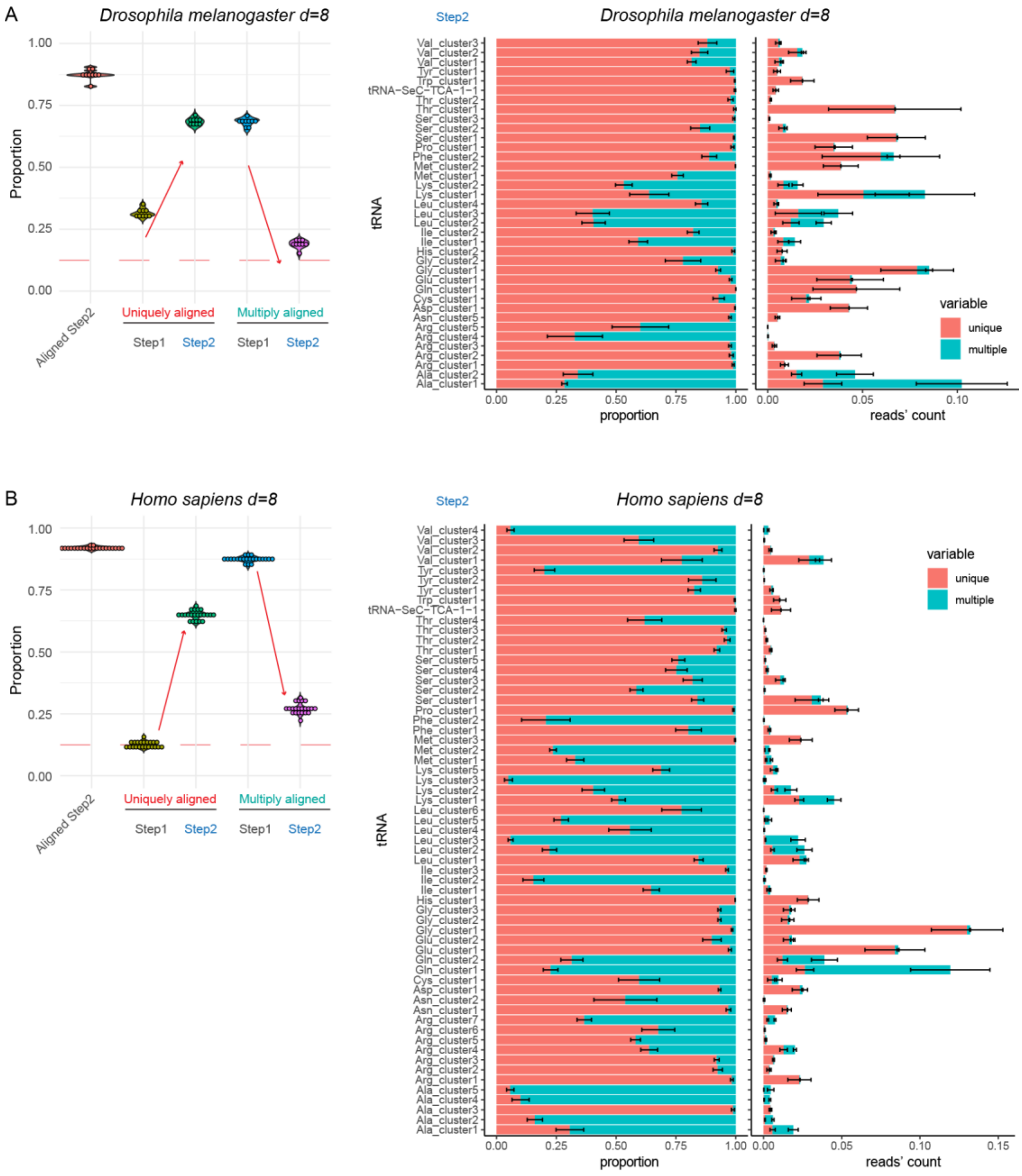

Figure S2 Alignment results for Non Duplicated (non redundant) tRNA reference (Step1) and optimized tRNA set (Step2) for *D. melanogaster* and *H. sapiens* references, maximal distance used is 8 substitutions. Boxplot on the left shows the proportion of tRNA sequencing reads aligned to Step2 reference ('Aligned Step2') and proportions of uniquely and multiply mapped reads at both steps. Red dashed line indicate 12.5% level. Increase of unique mapping and decrease of multiple mapping is shown by arrows. Barplots at the right represent unique and multiple mapping by tRNA species at Step2, in proportion to total and in absolute number of sequencing reads obtained by tRNA, expressed as proportion to total number of mapped reads. tRNAs showing excessive proportion of ambiguous mapping are shown in red.

```
D.melanogaster tRNALeu2 and tRNALeu3
Leu3 1>GTCAGGATGGCCGAGTGGTCTAAGGCGCTGCGTTCAGGTCGCAGTCTACTCTGTAGGCGTGGGTTCGAATCCCACTTCTGACA>83
Leu2 1>GTCAGGATGGCCGAGCGGTCTAAGGCGCCAGACTCAAGTTCTGGTCCTCTCTGAGGGCGTGGGTTCGAATCCCACTTCTGACA>83

H.sapiens tRNALeu2 and tRNALeu3
Leu3 1>GTCAGGATGGCCGAGCNGTCTNAAGGCGCTGCGTTCANNTCGCANNCTCC-NCTGGAGGCGTGGGTTCGAATCCCACTNNTGACA>84
Leu2 1>GTCAGGATGGCCGAGTGGTCT-AAGGCGCCAGACTCAAGTTCTGGTCTCCNNATGGAGGCGTGGGTTCGAATCCCACTTCTGACA>84

H.sapiens tRNATyr1, Tyr2 and and Tyr3
Tyr2 1>CCTTCAATAGTTCAGCTGGTAGAGCAGAGGACTATAGGTCCTTAGGTT-GCTGGTTCGATTCCAGCTTGAAGGA>73
Tyr3 1>TCTTCAATAGCTCAGCTGGTAGAGCGGAGGACTGTAGATTCTTAGG-T-GCTGGTTTGATTCCGACTTGGAGAG>72 minor

Tyr3 1>TCTTCAATAGCTCAGCTGGTAGAGCGGAGGACTGTAGATTCTTAGG-T-GCTGGTTTGATTCCGACTTGGAGAG>72 minor
Tyr1 1>CCTTCGATAGCTCAGNTGGTAGAGCGGAGGACTGTAGATCCTTAGG-TCGCTGGTTCGANTCCGGCTCGAAGGA>73

H.sapiens tRNAAla1 and tRNAVal3
Val3 1>GGGGGTGTAGCTCAGTGGTAGAGCGTATGCTTAACATTCATGAGGCTCTGGGTTCGATCCCCAGCACTTCCA>72
Ala1 1>GGGGGTGTAGCTCAGTGGTAGAGCGCNTGCTTNGCATGTATGAGGCCCCGGGTTCGATCCCCGGCACCTCCA>72
```

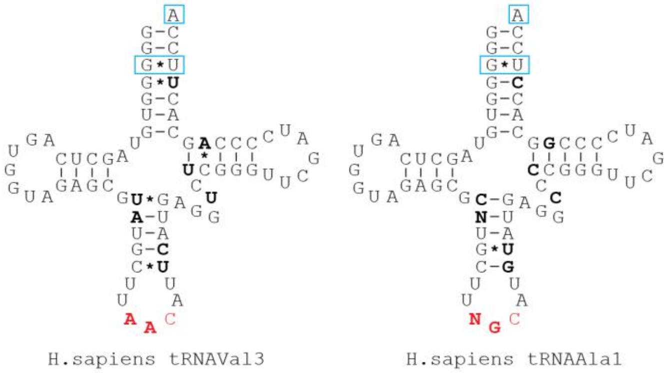

Figure S3 Sequences of tRNA species showing excessive ambiguous mapping for *D. melanogaster* and *H. sapiens* references. Non-identical nucleotides are in bold case, anticodon is in red and underlined.

Bottom – cloverleaf structures of *H. sapiens* $tRNA^{Val}_3$(AAC) and $tRNA^{Ala}_1$(NGC). Substitutions are in bold case. Anticodon is in red. Excessive number of mismatched nucleotides in $tRNA^{Val}_3$(AAC) stems may drive its instability (degradation) in vivo. Conserved identity elements for AlaRS aminoacylation (A73 and G3*U70) are boxed n bleu. 3'-CCA sequence not shown.

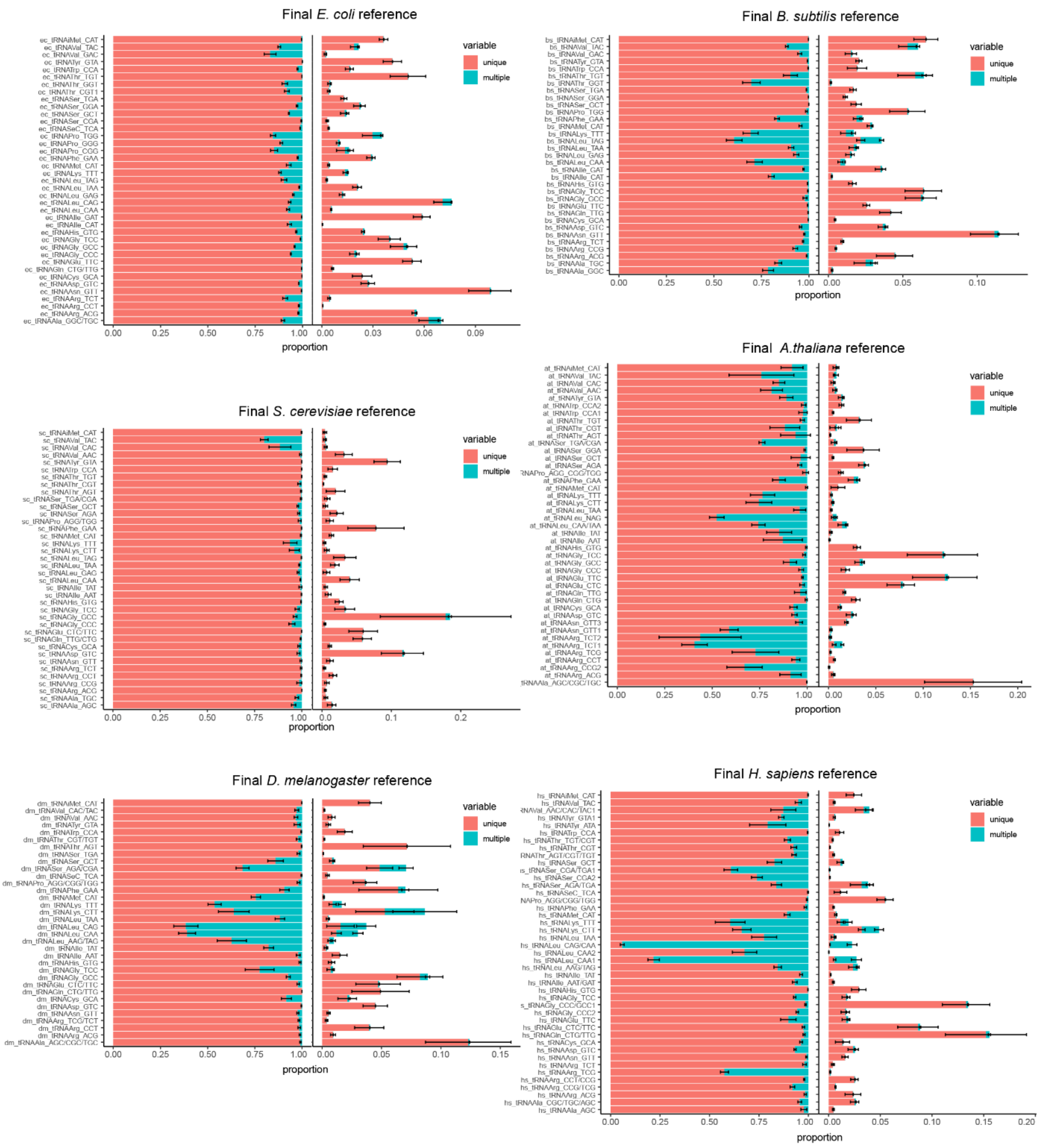

Figure S4 Barplots representing unique and multiple mapping by tRNA species for final manually curated tRNA references (Step 3), in proportion to total and in absolute number of sequencing reads obtained by tRNA, expressed as proportion to total number of mapped reads.

Table S1 Characteristics of datasets used in this study

	Rererence				
Number of raw reads for eache experimental dataset	tRNA_Step1	total_reads			
Full collection of RNA-aligned reads	tRNA_Step2	total_reads	aligned 1 time+aligned >1 time		
			Total raw teads	tRNA reads	% of tRNA reads
E.coli	Sample1	tRNA fraction	6263160	2740932	43,76%
	Sample2	tRNA fraction	7055237	3146981	44,60%
	Sample3	tRNA fraction	6761865	3090704	45,71%
	Sample4	tRNA fraction	6734371	2734340	40,60%
	Sample5	tRNA fraction	7069232	2859861	40,46%
	Sample6	tRNA fraction	6384822	2541778	39,81%
	Sample7	tRNA fraction	6913919	2717982	39,31%
	Sample8	tRNA fraction	7100802	2966758	41,78%
	Sample9	tRNA fraction	5942508	2464554	41,47%
B.subtilis	Sample1	total RNA	11850717	1082144	9,13%
	Sample2	total RNA	9905607	907391	9,16%
	Sample3	total RNA	8083896	344249	4,26%
	Sample4	total RNA	10314385	515336	5,00%
	Sample5	total RNA	10728232	618823	5,77%
	Sample6	total RNA	8411810	563490	6,70%
S.cerevisiae	Sample1	tRNA fraction	6122762	2493122	40,72%
	Sample2	tRNA fraction	3689248	1762221	47,77%
	Sample3	total RNA	7370764	342154	4,64%
	Sample4	tRNA fraction	5825861	1544905	26,52%
	Sample5	tRNA fraction	8271492	1418863	17,15%
	Sample6	total RNA	2185479	103817	4,75%
	Sample7	total RNA	2509444	140624	5,60%
	Sample8	total RNA	3721514	114760	3,08%
	Sample9	total RNA	4708804	314691	6,68%
A.thaliana	Sample1	total RNA	987701	41512	4,20%
	Sample2	total RNA	1253825	4088	0,33%
	Sample3	total RNA	1372257	4643	0,34%
	Sample4	total RNA	1167114	12066	1,03%
	Sample5	total RNA	1020172	50178	4,92%
	Sample6	total RNA	750254	50086	6,68%
	Sample7	total RNA	783698	2820	0,36%
	Sample8	total RNA	959869	32194	3,35%
	Sample9	total RNA	996683	3909	0,39%
D.melanogaster	Sample1	total RNA	8251871	132553	1,61%
	Sample2	total RNA	6870549	169509	2,47%

	Sample3	total RNA	8074747	182283	2,26%
	Sample4	total RNA	7095027	175671	2,48%
	Sample5	total RNA	7708891	91181	1,18%
	Sample6	total RNA	3773733	147751	3,92%
	Sample7	total RNA	5028137	133589	2,66%
	Sample8	total RNA	3868242	380404	9,83%
	Sample9	total RNA	4125326	108959	2,64%
H.sapiens	Sample1	total RNA	5709307	484384	8,48%
	Sample2	total RNA	7054479	1272210	18,03%
	Sample3	total RNA	6045177	1411462	23,35%
	Sample4	total RNA	7712830	983453	12,75%
	Sample5	total RNA	8075164	853727	10,57%
	Sample6	total RNA	5616878	1521167	27,08%
	Sample7	total RNA	7372670	819355	11,11%
	Sample8	total RNA	7736096	578414	7,48%
	Sample9	total RNA	4610880	503939	10,93%
	Sample10	total RNA	21143768	2102833	9,95%
	Sample11	total RNA	25065494	2563001	10,23%
	Sample12	total RNA	20896274	2321345	11,11%
	Sample13	total RNA	23307498	2936908	12,60%
	Sample14	total RNA	21629529	2435938	11,26%
	Sample15	total RNA	20399296	2024903	9,93%
	Sample16	total RNA	20384433	2390957	11,73%
	Sample17	total RNA	10588468	1058261	9,99%
	Sample18	total RNA	7750967	776801	10,02%
	Sample19	total RNA	8255650	630847	7,64%
	Sample20	total RNA	10175967	572478	5,63%
	Sample21	total RNA	9393861	600585	6,39%
	Sample22	total RNA	10174935	848865	8,34%

Detailed description of modifications in final tRNA references

Problematic cases

Escherichia coli

```
>Thr_cluster1 consensus sequence tRNA-Thr-CGT-1-1
GCCGATATAGCTCAGTTGGTAGAGCAGCGCATTCGTAATGCGAAGGTCGTAGGTTCGACTCCTATTATCGGCACCA
>Thr_cluster2 consensus sequence tRNA-Thr-CGT-2-1
GTAGTTAAAAATGCATTAACATCGCATTCGTAATGCGAAGGTCGTAGGTTCGACTCCTATTATCGGCACCA
>Thr_cluster3 consensus sequence tRNA-Thr-GGT-1-1|tRNA-Thr-GGT-2-1
GCTGATATNGCTCAGTTGGTAGAGCGCACCCTTGGTAAGGGTGAGGTCNNCAGTTCGANTCTGNNTATCAGCACCA
>Thr_cluster4 consensus sequence tRNA-Thr-TGT-1-1
GCCGACTTAGCTCAGTAGGTAGAGCAACTGACTTGTAATCAGTAGGTCACCAGTTCGATTCCGGTAGTCGGCACCA

Thr_cluster1 -- Matches:76; Mismatches:0; Gaps:0; Unattempted:0
Thr_cluster2 -- Matches:60; Mismatches:11; Gaps:5; Unattempted:0
Thr_cluster3 -- Matches:54; Mismatches:22; Gaps:0; Unattempted:0
Thr_cluster4 -- Matches:56; Mismatches:20; Gaps:0; Unattempted:0

* * * * * * *
Thr_cluster1 1>GCCGATATAGCTCAGTTGGTAGAGCAGCGCATTCGTAATGCGAAGGTCGTAGGTTCGACTCCTATTATCGGCACCA>76
Thr_cluster2 1>GTAGTTAAAAATGCATT-----AACATCGCATTCGTAATGCGAAGGTCGTAGGTTCGACTCCTATTATCGGCACCA>71
Thr_cluster3 1>GCTGATATNGCTCAGTTGGTAGAGCGCACCCTTGGTAAGGGTGAGGTCNNCAGTTCGANTCTGNNTATCAGCACCA>76
Thr_cluster4 1>GCCGACTTAGCTCAGTAGGTAGAGCAACTGACTTGTAATCAGTAGGTCACCAGTTCGATTCCGGTAGTCGGCACCA>76
```

Conclusion: Thr_cluster2 CGT=ec_tRNAThr_CGT2 is probable pseudogene, no U8, no G18/G19 in the D-loop (removed from the reference)

```
>Arg_cluster1 consensus sequence tRNA-Arg-ACG-1-1=tRNA-Arg-ACG-1-2=tRNA-Arg-ACG-1-3=tRNA-Arg-ACG-1-4
GCATCCGTAGCTCAGCTGGATAGAGTACTCGGCTACGAACCGAGCGGTCGGAGGTTCGAATCCTCCCGGATGCACCA
>Arg_cluster2 consensus sequence tRNA-Arg-CCG-1-1
GCGCCCGTAGCTCAGCTGGATAGAGCGCTGCCCTCCGGAGGCAGAGGTCTCAGGTTCGAATCCTGTCGGGCGCGCCA
>Arg_cluster3 consensus sequence tRNA-Arg-CCT-1-1
GTCCTCTTAGTTAAATGGATATAACGAGCCCCTCCTAAGGGCTAATTGCAGGTTCGATTCCTGCAGGGGACACCA
>Arg_cluster4 consensus sequence tRNA-Arg-TCT-1-1
GCGCCCTTAGCTCAGTTGGATAGAGCAACGACCTTCTAAGTCGTGGGCCGCAGGTTCGAATCCTGCAGGGCGCGCCA

Arg_cluster1 -- Matches:77; Mismatches:0; Gaps:0; Unattempted:0
Arg_cluster2 -- Matches:57; Mismatches:20; Gaps:0; Unattempted:0
Arg_cluster3 -- Matches:45; Mismatches:29; Gaps:4; Unattempted:0
Arg_cluster4 -- Matches:55; Mismatches:21; Gaps:2; Unattempted:0

* * * * * * *
>Arg_cluster1 1>GCATCCGTAGCTCAGCTGGATAGAGTACTCGGCTACGAA-CCGAGC-GGTCGGAGGTTCGAATCCTCCCGGATGCACCA>77
>Arg_cluster2 1>GCGCCCGTAGCTCAGCTGGATAGAGCGCTGCCCTCCGGA-GGCAGA-GGTCTCAGGTTCGAATCCTGTCGGGCGCGCCA>77
>Arg_cluster3 1>GTCCTCTTAG-TTAAATGGATATAACGAGCCCCTCCTAA---GGGCTAATTGCAGGTTCGATTCCTGCAGGGGACACCA>75
>Arg_cluster4 1>GCGCCCTTAGCTCAGTTGGATAGAGCAACGACCTTCTAAGTCGTG—GGCCGCAGGTTCGAATCCTGCAGGGCGCGCCA>77
```

Conclusion: Low-expressed Arg_cluster2=ec_tRNAArg_CCG shows identical regions with highly expressed Arg_cluster1 (thus Arg_cluster2 is removed from the reference)

Inosine-34 is known to be present in:

```
>ec_tRNAArg_ACG gcatccgtagctcagctggatagagtactcggctGcgaaccgagcggtcggaggttcgaatcctcccggatgcacca
```

Final E.coli reference 39 sequences : Final_Step3_Escherichia_coli_str_K-12_substr_MG1655__39seq_2020-11-27

Bacillus subtilis

```
>Arg_cluster1 consensus sequence tRNA-Arg-ACG-1-1=tRNA-Arg-ACG-1-2=tRNA-Arg-ACG-1-3=tRNA-Arg-ACG-1-4
GCGCCCGTAGCTCAATTGGATAGAGCGTTTGACTACGGATCAAAAGGTTAGGGGTTCGACTCCTCTCGGGCGCGCCA
>Arg_cluster2 consensus sequence tRNA-Arg-CCG-1-1
GCGCTCGTAGCTCAGTTGGATAGAGCGGTGGTTTCCGGTACCACGTCTGTCGGGGGTTCGAATCCCTCCGAGCGCG
>Arg_cluster3 consensus sequence tRNA-Arg-CCT-1-1
GCTCTAGTAGCACAGCGGATAGTGCAGCAGTTTCCTAAACTGCAGGTCGGGAGTTCGAATCTCTCCTAGAGCG
>Arg_cluster4 consensus sequence tRNA-Arg-TCT-1-1
GTCCCAGTAGCTCAGCTGGATAGAGCAACGGCCTTCTAAGCCGTCGGTCGGGAGTTCGAATCTCTCCTGGGACG

>Arg_cluster1 -- Matches:77; Mismatches:0; Gaps:0; Unattempted:0
>Arg_cluster2 -- Matches:55; Mismatches:19; Gaps:5; Unattempted:0
>Arg_cluster3 -- Matches:45; Mismatches:27; Gaps:6; Unattempted:0
>Arg_cluster4 -- Matches:47; Mismatches:27; Gaps:3; Unattempted:0

* * * * * * *
>Arg_cluster1 1>GCGCCCGTAGCTCAATTGGATAGAGCGTTTGACTACGG-ATCA-AAAGGTTAGGGGTTCGACTCCTCT-CGGGCGCGCCA>77
>Arg_cluster2 1>GCGCTCGTAGCTCAGTTGGATAGAGCGGTGGTTTCCGGTACCACGTCTGTCGGGGGTTCGAATCCCTC-CGAGCGCG~~~>76
>Arg_cluster3 1>GCTCTAGTAGCAC-AGCGGATAGTGCAGCAGTTTCCTA-AACT-GCAGGTCGGGAGTTCGAAT-CTCTCCTAGAGCG~~~>73
>Arg_cluster4 1>GTCCCAGTAGCTCAGCTGGATAGAGCAACGGCCTTCTA-AGCC-GTCGGTCGGGAGTTCGAAT-CTCTCCTGGGACG>74
```

Conclusion: Low-expressed Arg_cluster3=bs_tRNAArg_CCT shows identical regions with highly expressed Arg_cluster4 (thus Arg_cluster3 is removed from the reference)

```
>Leu_cluster3 consensus sequence tRNA-Leu-CAG-1-1
GCGGATGTGGCGGAATTGGCAGACGCGCTAGAATCAGGCTCTAGTGTCTTTACAGACGTGGGGGTTCAAGTCCCTTCATCCGCACCA
>Leu_cluster5 consensus sequence tRNA-Leu-TAG-1-1|tRNA-Leu-TAG-2-1
GCGGGTGTGGCGGAATTGGCAGACGCGCTAGACTTAGGATCTAGTGTCTTNANGACGTGGGGGTTCNAGTCCCTTCACCCGCA
```

>Leu_cluster3 -- Matches:75; Mismatches:8; Gaps:4; Unattempted:0
>Leu_cluster5 -- Matches:83; Mismatches:0; Gaps:0; Unattempted:0

* * * * * * * *
>Leu_cluster3 1>GCGGATGTGGCGGAATTGGCAGACGCGCTAGAATCAGGCTCTAGTGTCTTTACAGACGTGGGGTTCAAGTCCCTTCATCCGCACCA
>Leu_cluster5 1>GCGGGTGTGGCGGAATTGGCAGACGCGCTAGACTTAGGATCTAGTGTCTTNA-NGACGTGGGGTTCNAGTCCCTTCACCCGCA

Conclusion: Very high level of identity between two tRNAs Leu_cluster3=bs_tRNALeu_CAG is removed from the reference

Inosine-34 is known to be present in:

>bs_tRNAArg_ACG gcgcccgtagctcaattggatagagcgtttgactGcggatcaaaaggttaggggttcgactcctctcgggcgcgcca

Saccharomyces cerevisiae
No manual adaptations required

Inosine-34 is known to be present in:
>sc_tRNAAla_AGC gggcgtgtggcgtagtcggtagcgcgctcccttGgcatgggagaggtctccggttcgattccggactcgtccacca
>sc_tRNAArg_ACG ttcctcgtggcccaatggtcacggcgtctggctGcgaaccagnagattccaggttcnagtcctggcggggaagcca
>sc_tRNAIle_AAT ggtctcttggcccagttggttaaggcaccgtgctGataacgcggggatcagcggttcgatcccgctagagaccacca
>sc_tRNASer_AGA ggcaacttggccgagtggttaaggcgaaagattGgaaatcttttgggctttgncccgcgcaggttcgagtcctgcagttgtcgcca
>sc_tRNAThr_AGT gcttctatggccaagttggtaaggcgccacactGgtaatgtggagatcatcggttcaaatccgattggaagcacca
>sc_tRNAVal_AAC ggtttcgtggtctagtcggttatggcatctgcttGacacgcagaacgtccccagttcgatcctgggcgaaatcncca

Arabidopsis thaliana
>Arg_cluster1 consensus sequence tRNA-Arg-ACG-1-1=tRNA-Arg-ACG-5-1=tRNA-Arg-ACG-5-2|tRNA-Arg-ACG-2-1=tRNA-Arg-ACG-3-1=tRNA-Arg-ACG-3-2|tRNA-Arg-ACG-6-1=tRNA-Arg-ACG-6-2|tRNA-Arg-ACG-4-1
GACTCCATGGCCCAATGGATAAGGCGCTGGTCTACGAAACCAGAGATTCTGGGTTCGATCCCCAGTGGAGTCG
>Arg_cluster2 consensus sequence tRNA-Arg-TCG-1-1=tRNA-Arg-TCG-1-2=tRNA-Arg-TCG-1-3|tRNA-Arg-TCG-2-1=tRNA-Arg-TCG-2-2=tRNA-Arg-TCG-2-3
GACCGCATAGCGCAGTGGATTAGCGCGTNTGACTTCGGATCANAAGGTCGTGGGTTCGACTCCCACTGTGGTCG
>Arg_cluster3 consensus sequence tRNA-Arg-TCT-1-1=tRNA-Arg-TCT-5-1=tRNA-Arg-TCT-5-2|tRNA-Arg-TCT-2-1=tRNA-Arg-TCT-7-1=tRNA-Arg-TCT-8-1|tRNA-Arg-TCT-3-1=tRNA-Arg-TCT-4-1
GCACCCGTGGCCTAATGGATAAGGCGTTTGACTTCTAATCAAACGATTGTGGGTTCGAGTCCCACCGGGTGTG
>Arg_cluster4 consensus sequence tRNA-Arg-CCG-3-1=tRNA-Arg-CCG-3-2|tRNA-Arg-CCG-2-1
GNNNGCGTGGCCTAATGGATAAGGCGCTCGCCTCCGNAGCGGGAGATTGTGGGTTCGANTCCCANCGNGNNCG
>Arg_cluster5 consensus sequence tRNA-Arg-CCT-1-1=tRNA-Arg-CCT-1-2=tRNA-Arg-CCT-1-3=tRNA-Arg-CCT-1-4=tRNA-Arg-CCT-1-5=tRNA-Arg-CCT-2-1|tRNA-Arg-CCT-3-1
GCGCCTGTAGCTCAGTGGATAGAGCGTCTGTTTCCTAAGCAGAANGTCGNAGGTTCGACCCCTNCCTGGCGCG
>Arg_cluster6 consensus sequence tRNA-Arg-TCT-6-1
GCGCTCGTGGCCCAATGGATAAGGCGTCTGACTTCTAATCAGACGATTGTGGGTTCGATCCCCACCGAGCGTG
>Arg_cluster7 consensus sequence tRNA-Arg-CCG-1-1
GATCCCATAGCGGAGTGGATATCGCGTTAGACTCCGAATCTAAAGGTCGTGGGTTCGATTCCCACTGGGATCA

>Arg_cluster1 1>GACTCCATGGCCCAATGGATAAG-GCGCTGGTCTACGAAACCAGAGATTCTGGGTTCGATCCC-CAGTG-GAGTCG>73
>Arg_cluster2 1>GACCGCATAGCGCAGTGGATTAGCGCGTNTGACTTCGGATCANAAGGTCGTGGGTTCGACTCC-CACTG-TGGTCG>74
>Arg_cluster3 1>GCACCCGTGGCCTAATGGATAAG-GCGTTTGACTTCTAATCAAACGATTGTGGGTTCGAGTCC-CACCG-GGTGTG>73
>Arg_cluster4 1>GNNNGCGTGGCCTAATGGATAAG-GCGCTCGCCTCCGNAGCGGGAGATTGTGGGTTCGANTCC-CANCG-NGNNCG>73
>Arg_cluster5 1>GCGCCTGTAGCTCAGTGGATAGA-GCGTCTGTTTCCTAAGCAGAANGTCGNAGGTTCGACCCCTNCCTG-GCG-CG>73
>Arg_cluster6 1>GCGCTCGTGGCCCAATGGATAAG-GCGTCTGACTTCTAATCAGACGATTGTGGGTTCGATCCC-CACCGAGCGT-G>73
>Arg_cluster7 1>GATCCCATAGCGGAGTGGATATC-GCGTTAGACTCCGAATCTAAAGGTCGTGGGTTCGATTCC-CACTG-GGATCA>73

Conclusion: Low expressed Arg_cluster4=at_tRNAArg_CCG1 is highly similar to Arg_cluster3 and thus can be removed

>Asn_cluster1 consensus sequence tRNA-Asn-GTT-3-1=tRNA-Asn-GTT-3-2=tRNA-Asn-GTT-3-3=tRNA-Asn-GTT-3-4=tRNA-Asn-GTT-3-5=tRNA-Asn-GTT-3-6|tRNA-Asn-GTT-1-1|tRNA-Asn-GTT-2-1|tRNA-Asn-GTT-8-1
GCTGGAATAGCTCAGTTGGTTAGAGCGTGTGGCTGTTAACCACAAGGTCGGAGGTTCGANCCCTCCTTCTAGCG
>Asn_cluster2 consensus sequence tRNA-Asn-GTT-4-1=tRNA-Asn-GTT-4-2=tRNA-Asn-GTT-4-3=tRNA-Asn-GTT-4-4|tRNA-Asn-GTT-5-1
GCTGGAGTAGCTCAGTTGGTTAGAGCGTGTGGCTGTTAACCACAAGGTCAGAGGTTCGACCCCTNTCTCTAGCG
>Asn_cluster3 consensus sequence tRNA-Asn-GTT-7-1
TCCTCAGTAGCTCAGTGGTAGAGCGGTCGGCTGTTAACTGATTGGTCGTAGGTTCAAATCCTACTTGGGGAG
>Asn_cluster4 consensus sequence tRNA-Asn-GTT-9-1
GCTGGAATAGCTCAGTAGGTTTAGAGCGTGTGGCTGTTAACCTCAAGGTCGGAGGTTCGACCCCTCCTTTCAGCG

1>GCTGGAATAGCTCAGTTGG-TTAGAGCGTGTGGCTGTTAACCACAAGGTCGGAGGTTCGANCCCTCCTTCTAGCG>74
1>GCTGGAGTAGCTCAGTTGG-TTAGAGCGTGTGGCTGTTAACCACAAGGTCAGAGGTTCGACCCCTNTCTCTAGCG>74
1>GCTGGAATAGCTCAGTAGGTTTAGAGCGTGTGGCTGTTAACCTCAAGGTCGGAGGTTCGACCCCTCCTTTCAGCG>75

Conclusion: Merge Asn_cluster1=at_tRNAAsn_GTT1 and Asn_cluster2=at_tRNAAsn_GTT2, Asn_cluster4=at_tRNAAsn_GTT4 removed
1>GCTGGAATAGCTCAGTTGG-TTAGAGCGTGTGGCTGTTAACCACAAGGTCGGAGGTTCGANCCCTCCTTCTAGCG>74
1>GCTGGAGTAGCTCAGTTGG-TTAGAGCGTGTGGCTGTTAACCACAAGGTCAGAGGTTCGACCCCTNTCTCTAGCG>74
Consensus 1>gctggantagctcagttgg-ttagagcgtgtggctgttaaccacaaggtcngaggttcganccctnnntctagcg>74

>Leu_cluster1 consensus sequence tRNA-Leu-AAG-1-1=tRNA-Leu-AAG-1-10=tRNA-Leu-AAG-1-2=tRNA-Leu-AAG-1-3=tRNA-Leu-AAG-1-4=tRNA-Leu-AAG-1-5=tRNA-Leu-AAG-1-6=tRNA-Leu-AAG-1-7=tRNA-Leu-AAG-1-8=tRNA-Leu-AAG-1-9=tRNA-Leu-AAG-2-1|tRNA-Leu-CAG-1-1=tRNA-Leu-CAG-1-2=tRNA-Leu-CAG-1-3
GTNNANATGGCCGAGTTGGTCTAAGGCGCCAGNTTNAGGTNCTGGTCCGAAAGGGCGTGGGTTCAAATCCCACTNTNNACA
>Leu_cluster2 consensus sequence tRNA-Leu-TAG-1-1=tRNA-Leu-TAG-1-2=tRNA-Leu-TAG-1-3=tRNA-Leu-TAG-1-4|tRNA-Leu-TAG-2-1=tRNA-Leu-TAG-2-2=tRNA-Leu-TAG-2-3|tRNA-Leu-TAG-3-1=tRNA-Leu-TAG-3-2
GACAGTTTGGCCGAGTGGTCTAAGGCGCCAGATTTAGGCTCTGGTCCGAAAGGGCGTGGGTTCAAATCCCACAGCTGTCA

```
>Leu_cluster3 consensus sequence tRNA-Leu-CAA-1-1=tRNA-Leu-CAA-1-2=tRNA-Leu-CAA-1-3=tRNA-Leu-CAA-3-1|tRNA-Leu-CAA-2-1=tRNA-Leu-CAA-2-2=tRNA-Leu-CAA-2-3|tRNA-Leu-CAA-5-1=tRNA-Leu-CAA-5-2|tRNA-Leu-CAA-4-1|tRNA-Leu-TAA-1-1
GTCAGGATGGCCGAGTGGTCTAAGGCGCCAGACTCAAGTTCTGGTCCTCGTAAGAGGGCGTGGGTTCAAATCCCACTTCTGACA
>Leu_cluster4 consensus sequence tRNA-Leu-TAA-2-1=tRNA-Leu-TAA-2-2=tRNA-Leu-TAA-2-3=tRNA-Leu-TAA-3-1=tRNA-Leu-TAA-3-2
gcaggtttgcccgagtggttaaggggaagacttaagttcttctgcacataagtgcgcgtgggttcgaaccccacagcctgca
>Leu_cluster5 consensus sequence tRNA-Leu-AAG-4-1
gggcatttggtctagtggtttgattttcgcttaaggtgcgagaggtcccgagttcaattctcagaatgcccc
```

```
Leu_cluster1 GTNNANATGGCCGAGTTGGTCTAAGGCGCCAGNTTNAGGTNCTGGTCC----GAAAGGGCGTGGGTTCAAATCCCACTNTNNACA
Leu_cluster2 GACAGTTTGGCCGAGT-GGTCTAAGGCGCCAGATTTAGGCTCTGGTCC----GAAAGGGCGTGGGTTCAAATCCCACAGCTGTCA
Leu_cluster3 GTCAGGATGGCCGAGT-GGTCTAAGGCGCCAGACTCAAGTTCTGGTCCTCGTAAGAGGGCGTGGGTTCAAATCCCACTTCTGACA
Leu_cluster4 gcaggtttgcccgagtggttaaggggaagacttaagttcttctgcacataagtgcgcgtgggttcgaaccccacagcctgca
Leu_cluster5 gggcatttggtctagtggtttgattttcgcttaaggtgcgagaggtcccgagttcaattctcagaatgcccc
```

Conclusion: Sequence Leu_cluster5=at_tRNALeu_AAG2 too short, unique gene tRNA-Leu-AAG-4-1 (pseudogene?), to remove

```
Leu_cluster1 GTNNANATGGCCGAGTTGGTCTAAGGCGCCAGNTTNAGGTNCTGGTCC----GAAAGGGCGTGGGTTCAAATCCCACTNTNNACA
Leu_cluster2 GACAGTTTGGCCGAGT-GGTCTAAGGCGCCAGATTTAGGCTCTGGTCC----GAAAGGGCGTGGGTTCAAATCCCACAGCTGTCA
Leu_cluster3 GTCAGGATGGCCGAGT-GGTCTAAGGCGCCAGACTCAAGTTCTGGTCCTCGTAAGAGGGCGTGGGTTCAAATCCCACTTCTGACA
```

Conclusion: Three tRNAs are very similar, so keep Leu_cluster3 (seems to be major) and collaps Leu_cluster 1 and 2 in one

```
Leu_cluster1 GTNNANATGGCCGAGTTGGTCTAAGGCGCCAGNTTNAGGTNCTGGTCC----GAAAGGGCGTGGGTTCAAATCCCACTNTNNACA
Leu_cluster2 GACAGTTTGGCCGAGT-GGTCTAAGGCGCCAGATTTAGGCTCTGGTCC----GAAAGGGCGTGGGTTCAAATCCCACAGCTGTCA
consensus    gnnnnnntggccgagttggtctaaggcgccagnttnaggnnctggtcc----gaaagggcgtgggttcaaatcccacnnnnnnca
```

Inosine-34 is most likely present in (no data in tRNAdb or MODOMICS):

```
>at_tRNAAla_AGC_CGC_TGC ggggatgtagctcatatggtagagcgctcgcttNgcatgcgagaggcacggggttcgattccccgcatctccacca
>at_tRNAArg_ACG       gactccatggcccaatggataaggcgctggtctGcgaaaccagagattctgggttcgatccccagtggagtcgcca
>at_tRNAIle_AAT       ggccnattagctcagttggttagagcgtcgtgctGataacgcgaaggtcncaggttcganncctgnatnggccacca
>at_tRNAPro_AGG_CGG_TGG gggcatttggtctagtggtatgattctcgcttNgggtgcgagaggtcccgagttcgattctcggaatgcccccca
>at_tRNASer_AGA       gtggacgtgccggagtggttatcgggcatgactGgaaatcatgtgggctttgcccgcgcaggttcgaatcctgccgttnacgcca
>at_tRNAThr_AGT       gctntcntagctcagttggttagagcacccgtttGgtaagcgggaggtcttgagttcaactctcaanganagcacca
>at_tRNAVal_AAC       ggtttcgtggtgtagttggttatcacgtcagtctGacacactnaaggtctccggttcgaacccgggcgaagccacca
N are NOT replaced
```

Drosophila melanogaster

```
>Lys_cluster2 consensus sequence tRNA-Lys-TTT-2-1=tRNA-Lys-TTT-2-2=tRNA-Lys-TTT-2-3=tRNA-Lys-TTT-2-4=tRNA-Lys-TTT-2-5|tRNA-Lys-TTT-1-1
GCCCGGNTAGCTCAGTCGGTAGAGCATTGGACTTTTAATCCAAGGGTCCAGGGTTCAAGTCCCTGNTCGGGCGcca
>Lys_cluster1 consensus sequence tRNA-Lys-CTT-1-1=tRNA-Lys-CTT-1-10=tRNA-Lys-CTT-1-11=tRNA-Lys-CTT-1-12=tRNA-Lys-CTT-1-13=tRNA-Lys-CTT-1-2=tRNA-Lys-CTT-1-3=tRNA-Lys-CTT-1-4=tRNA-Lys-CTT-1-5=tRNA-Lys-CTT-1-6=tRNA-Lys-CTT-1-7=tRNA-Lys-CTT-1-8=tRNA-Lys-CTT-1-9
gcccggctagctcagtcggtagagcatgagactcttaatctcagggtcgtgggttcgagccccacgttgggcgCCA
```

```
>Lys_cluster1      1>gcccggctagctcagtcggtagagcatgagactcttaatctcagggtcgtgggttcgagccccacgttgggcgCCA>76
>Lys_cluster2      1>GCCCGGNTAGCTCAGTCGGTAGAGCATTGGACTTTTAATCCAAGGGTCCAGGGTTCAAGTCCCTGNTCGGGCGcca>76
```

Conclusion: 14 different nucleotides, but 3'-end is common, keep both sequences, Lys_TTT seems to be minor tRNA

```
>Leu_cluster2 consensus sequence tRNA-Leu-CAA-1-1=tRNA-Leu-CAA-2-1=tRNA-Leu-CAA-2-2=tRNA-Leu-CAA-2-3
gtcaggatggccgagcggtctaaggcgccagactcaagttctggtcctctgagggcgtgggttcgaatcccacttctgacaCCA
>Leu_cluster3 consensus sequence tRNA-Leu-CAG-1-1=tRNA-Leu-CAG-1-2=tRNA-Leu-CAG-1-3=tRNA-Leu-CAG-1-4=tRNA-Leu-CAG-1-5=tRNA-Leu-CAG-1-6=tRNA-Leu-CAG-1-7=tRNA-Leu-CAG-1-8
gtcaggatggccgagtggtctaaggcgctgcgttcaggtcgcagtctactctgtaggcgtgggttcgaatcccacttctgacaCCA
```

```
>Leu_cluster2 1>gtcaggatggccgagcggtctaaggcgccagactcaagttctggtcctctgagggcgtgggttcgaatcccacttctgacaCCA>86
>Leu_cluster3 1>gtcaggatggccgagtggtctaaggcgctgcgttcaggtcgcagtctactctgtaggcgtgggttcgaatcccacttctgacaCCA>86
```

Conclusion: Two tRNAs are too similar at the 5'- and 3'-ends, keep both

```
>Arg_cluster4 consensus sequence tRNA-Arg-TCT-1-1|tRNA-Arg-TCT-2-1
GNCCCTTTNGCGCANNGGATAGCGCGTTGGACTTCTAATCCAAAGGTNGCGGGTTCGATNCCCGCAAGGGNTGcca
```

Conclusion: Arg_cluster4=dm_tRNAArg_TCT is low expressed, contains too many N, may be similar with Arg_cluster3, thus removed

Inosine-34 is known to be present in:

```
>dm_tRNASer_AGA_CGA   gcagtcgtggccgagcggttaaggcgtctgactGgaaatcagattccctctgggagcgtaggttcgaatcctaccggctgcgcca
>dm_tRNAVal_AAC         gtttccgtggtgtagnggttatcacatcngcctGacacgcngaaggcccccggttcnatcccgggcggaaacacca
```

And is most likely present in (no data in tRNAdb or MODOMICS):

```
>dm_tRNAAla_AGC_CGC_TGC ggggatgtagctcagtggtagagcgctcgcttNgcatgtgagaggtcccgggttcgatccccggcatctccacca
>dm_tRNAArg_ACG         ggtcctgtggcgcaatggataacgcgtctgactGcggatcagaagattccaggttcgactcctggcaggatcgcca
>dm_tRNAIle_AAT         ggcccattagctcagttggttagagcgtcgtgctGataacgcgaaggtcgcgggttcgatcccctcatgggccacca
>dm_tRNALeu_AAG_TAG   ggnagcgtggccgagcggtctaaggcgctggtttNaggcaccagtcnncnnnnnggcgtgggttcgaatcccaccgctgncacca
>dm_tRNAPro_AGG_CGG_TGG ggctcgttggtctaggggtatgattctcgcttcgggtgcgagaggtcccgggttcaaatcccggacgagcccccca
>dm_tRNAThr_AGT         ggcgccgtggcttagttggttaaagcgcctgtctGgtaaacaggagatcgtgagttcgaatntcgccggggcctcca
```

Homo sapiens

```
>Leu_cluster2 consensus sequence tRNA-Leu-CAA-1-1=tRNA-Leu-CAA-1-2|tRNA-Leu-CAA-2-1=tRNA-Leu-CAA-3-1=tRNA-Leu-CAA-4-1
GTCAGGATGGCCGAGTGGTCTAAGGCGCCAGACTCAAGTTCTGGTCTCCNNATGGAGGCGTGGGTTCGAATCCCACTTCTGACAcca
>Leu_cluster3 consensus sequence tRNA-Leu-CAG-1-1=tRNA-Leu-CAG-1-2=tRNA-Leu-CAG-1-3=tRNA-Leu-CAG-1-4=tRNA-Leu-CAG-1-5=tRNA-Leu-CAG-1-6=tRNA-Leu-CAG-1-7=tRNA-Leu-CAG-2-1=tRNA-Leu-CAG-2-2|tRNA-Leu-CAA-6-1
GTCAGGATGGCCGAGCNGTCTNAAGGCGCTGCGTTCANNTCGCANNCTCCNCTGGAGGCGTGGGTTCGAATCCCACTNNTGACAcca
```

```
>Leu_cluster2 1>GTCAGGATGGCCGAGTGGTCT-AAGGCGCCAGACTCAAGTTCTGGTCTCCNNATGGAGGCGTGGGTTCGAATCCCACTTCTGACA>
>Leu_cluster3 1>GTCAGGATGGCCGAGCNGTCTNAAGGCGCTGCGTTCANNTCGCANNCTCC-NCTGGAGGCGTGGGTTCGAATCCCACTNNTGACA>84
```
Conclusion: Too similar at the 5’- and 3’-ends, keep both

```
>Tyr_cluster1 consensus sequence tRNA-Tyr-GTA-1-1=tRNA-Tyr-GTA-2-1|tRNA-Tyr-GTA-3-1=tRNA-Tyr-GTA-6-1|tRNA-Tyr-GTA-4-1=tRNA-Tyr-GTA-5-1=tRNA-Tyr-GTA-5-2=tRNA-Tyr-GTA-5-3=tRNA-Tyr-GTA-5-4=tRNA-Tyr-GTA-5-5|tRNA-Tyr-GTA-7-1|tRNA-Tyr-GTA-8-1
CCTTCGATAGCTCAGNTGGTAGAGCGGAGGACTGTAGATCCTTAGGTCGCTGGTTCGANTCCGGCTCGAAGGAcca
>Tyr_cluster2 consensus sequence tRNA-Tyr-ATA-1-1
ccttcaatagttcagctggtagagcagaggactataggtccttaggttgctggttcgattccagcttgaaggaCCA
>Tyr_cluster3 consensus sequence tRNA-Tyr-GTA-9-1
tcttcaatagctcagctggtagagcggaggactgtagattcttaggtgctggtttgattccgacttggagagCCA
```

```
>Tyr_cluster2 1>CCTTCAATAGTTCAGCTGGTAGAGCAGAGGACTATAGGTCCTTAGGTT-GCTGGTTCGATTCCAGCTTGAAGGA>73
>Tyr_cluster3 1>TCTTCAATAGCTCAGCTGGTAGAGCGGAGGACTGTAGATTCTTAGG-T-GCTGGTTTGATTCCGACTTGGAGAG>72 minor
```

```
>Tyr_cluster3 1>TCTTCAATAGCTCAGCTGGTAGAGCGGAGGACTGTAGATTCTTAGG-T-GCTGGTTTGATTCCGACTTGGAGAG>72 minor
>Tyr_cluster1 1>CCTTCGATAGCTCAGNTGGTAGAGCGGAGGACTGTAGATCCTTAGG-TCGCTGGTTCGANTCCGGCTCGAAGGA>73
```
Conclusion: Low expressed Tyr_cluster3 is removed

```
>Ala_cluster1 consensus sequence tRNA-Ala-CGC-1-1=tRNA-Ala-CGC-2-1=tRNA-Ala-TGC-2-1=tRNA-Ala-TGC-3-1=tRNA-Ala-TGC-3-2|tRNA-Ala-TGC-5-1=tRNA-Ala-TGC-7-1|tRNA-Ala-AGC-2-1=tRNA-Ala-AGC-2-2=tRNA-Ala-AGC-3-1=tRNA-Ala-AGC-7-1|tRNA-Ala-AGC-1-1|tRNA-Ala-CGC-3-1|tRNA-Ala-CGC-4-1|tRNA-Ala-TGC-1-1|tRNA-Ala-TGC-4-1|tRNA-Ala-TGC-6-1|tRNA-Ala-AGC-4-1|tRNA-Ala-AGC-5-1|tRNA-Ala-AGC-6-1
GGGGGTGTAGCTCAGTGGTAGAGCGCNTGCTTNGCATGTATGAGGCCCCGGGTTCGATCCCCGGCACCTCCAcca
>Val_cluster3 consensus sequence tRNA-Val-AAC-6-1
gggggtgtagctcagtggtagagcgtatgcttaacattcatgaggctctgggttcgatccccagcacttccaCCA
```

```
>Ala_cluster1 1>GGGGGTGTAGCTCAGTGGTAGAGCGCNTGCTTNGCATGTATGAGGCCCCGGGTTCGATCCCCGGCACCTCCA>72
>Val_cluster3 1>GGGGGTGTAGCTCAGTGGTAGAGCGTATGCTTAACATTCATGAGGCTCTGGGTTCGATCCCCAGCACTTCCA>72
```
Conclusion: Val_cluster3 is removed

Inosine-34 is known to be present in:

```
>hs_tRNAAla_CGC_TGC_AGC gggggtgtagctcagtggtagagcgcntgcttNgcatgtatgaggccccgggttcgatccccggcacctccacca
>hs_tRNAAla_AGC         ggggaattagctcaagtggtagagcgctcgcttGgcatgcgagaggtagcgggatcgatgcccgcattctccacca
```

And is most likely present in (no data in tRNAdb or MODOMICS):

```
>hs_tRNAArg_ACG     gggccagtggcgcaatggataacgcgtctgactGcggatcagaagattctaggttcgactcctggctggctcgcca
>hs_tRNAIle_AAT_GAT ggccggttagctcagttggttagagcgtggtgctGataacgccaaggtcgcgggttcgatccccgtacnggccacca
>hs_tRNALeu_AAG_TAG ggtagcgtggccgagcggtctaaggcgctggattGaggctccagtctcttcgggggcgtgggttcgaatcccaccgctgccacca
>hs_tRNAPro_AGG_CGG_TGG ggctcgttggtctaggggtatgattctcgcttNgggtgcgagaggtcccgggttcaaatcccggacgagcccca
>hs_tRNASer_AGA_TGA gtagtcgtggccgagtggttaaggcgatggactGgaaatccattggggtntccccgcgcaggttcgaatcctgccgactacgcca
>hs_tRNAThr_AGT_CGT_TGT ggctcngtggcttagttggttaaagcgcctgtctGgtaaacaggagatcctgggttcgaatcccagcggggcctcca
>hs_tRNATyr_ATA     ccttcaatagttcagctggtagagcagaggactGtaggtccttaggttgctggttcgattccagcttgaaggacca
>hs_tRNAVal_AAC_CAC_TAC1 gtttccgtagtgtagtggttatcacgttcgcctCacacgcgaaaggtccccggttcgaaaccgggcggaaacacca
```
